# Photoreceptor-targeted extracellular vesicles-mediated delivery of Cul7 siRNA for retinal degeneration therapy

**DOI:** 10.7150/thno.99484

**Published:** 2024-08-12

**Authors:** Dong Guo, Yuntong Sun, Junqi Wu, Linchao Ding, Yiwen Jiang, Yadong Xue, Yongjun Ma, Fengtian Sun

**Affiliations:** 1 Department of Clinical Laboratory, Jinhua Central Hospital, Teaching Hospital of Mathematical Medicine College, Zhejiang Normal University, Jinhua 321000, Zhejiang, China.; 2 Department of Orthopedic Surgery, Center for Orthopedic Surgery, The Third Affiliated Hospital of Southern Medical University, Guangzhou 510630, Guangdong, China.

**Keywords:** Extracellular vesicles, Retinal degeneration, Photoreceptors, Cul7, Ferroptosis

## Abstract

**Rationale:** Photoreceptor loss is a primary pathological feature of retinal degeneration (RD) with limited treatment strategies. RNA interference (RNAi) has emerged as a promising method of gene therapy in regenerative medicine. However, the transfer of RNAi therapeutics to photoreceptors and the deficiency of effective therapeutic targets are still major challenges in the treatment of RD.

**Methods:** In this study, photoreceptor-derived extracellular vesicles (PEVs) conjugated with photoreceptor-binding peptide MH42 (PEVs^MH42^) were prepared using the anchoring peptide CP05. Transcriptome sequencing was applied to investigate the potential therapeutic target of RD. We then engineered PEVs^MH42^ with specific small-interfering RNAs (siRNAs) through electroporation and evaluated their therapeutic efficacy in N-methyl-N-nitrosourea (MNU)-induced RD mice and Pde6β^rd1/rd1^ mutant mice.

**Results:** PEVs^MH42^ were selectively accumulated in photoreceptors after intravitreal injection. Cullin-7 (Cul7) was identified as a novel therapeutic target of RD. Taking advantage of the established PEVs^MH42^, siRNAs targeting Cul7 (siCul7) were efficiently delivered to photoreceptors and consequently blocked the expression of Cul7. Moreover, suppression of Cul7 effectively protected photoreceptors to alleviate RD both in MNU-induced mouse model and Pde6β^rd1/rd1^ mutant mouse model. Mechanistically, PEVs^MH42^ loaded with siCul7 (PEVs^MH42^-siCul7)-induced Cul7 downregulation was responsible for preventing Cul7-mediated glutathione peroxidase 4 (Gpx4) ubiquitination and degradation, resulting in the inhibition of photoreceptor ferroptosis.

**Conclusions:** In summary, PEVs^MH42^-siCul7 attenuate photoreceptor ferroptosis to treat RD by inhibiting Cul7-induced ubiquitination of Gpx4. Our study develops a PEVs-based platform for photoreceptor-targeted delivery and highlights the potential of PEVs^MH42^-siCul7 as effective therapeutics for RD.

## Introduction

Retinal degeneration (RD) is a leading cause of irreversible blindness worldwide 1. The common forms of RD mainly include retinitis pigmentosa, age-related macular degeneration, and retinal dystrophy 2. The pathological photoreceptor loss is considered an essential contributor to vision decline in patients with RD 3. Although significant efforts have been made to establish protective strategies such as gene therapy, anti-apoptotic drug injection, stem/progenitor cell transplantation, and retinal prosthesis devices, unfavorable side effects and unsatisfactory efficacy remain issues 4. Therefore, the elucidation of molecular mechanism underlying photoreceptor loss and the development of novel treatment methods for RD are urgently required.

RNA interference (RNAi) therapeutics such as small-interfering RNAs (siRNAs) and microRNAs represent a novel approach for the treatment of refractory diseases by directly targeting pathogenic genes 5. For example, the siRNAs targeting AEBP1 were reported to prevent retinal fibrosis in diabetic retinopathy 6. However, the delivery of RNAi therapeutics to specific tissues or cell types is still a major challenge. Current available nanocarriers mainly include liposomes, dendrimers, and calcium phosphate nanoparticles, whereas these synthetic vectors have many limitations such as immunogenicity, cytotoxicity, poor stability, and rapid clearance from circulation 7. Thus, it is important to establish safer and more effective transport strategies for RNAi therapeutics.

Extracellular vesicles (EVs) are nanoscale membrane particles derived from living cells in a constitutive or inducible manner 8. EVs naturally serve as intercellular messengers by transferring bioactive cargos including nucleic acids, proteins, and lipids to distal or nearby cells 9. Increasing evidence has revealed the potential of EVs as efficient and feasible drug delivery tools for treating various diseases 10, 11. Compared with synthetic nanocarriers, the natural origin of EVs enables them to escape phagocytosis, extend the half-life of drugs, and exhibit low immunogenicity 12. EVs also possess many intrinsic properties such as stability, biocompatibility, and biological barrier permeability 13. Moreover, the lipid bilayer membranes of EVs can be modified with peptides or functional ligands to further enhance tissue-specific targeting 14. The effective transportation of therapeutic agents by engineered EVs has attracted considerable attention in the field of tissue regeneration. Therefore, EV-based delivery platforms are promising candidates for photoreceptor-targeted RD therapy.

In this study, based on the anchor peptide CP05-mediated interaction with the EV marker CD63, photoreceptor-derived EVs (PEVs) were modified with the MH42 peptide, which can target photoreceptors 15. Then, transcriptome sequencing was performed to explore the potential therapeutic target of RD. We revealed that PEVs conjugated with MH42 peptide (PEVs^MH42^) allowed for an efficient delivery of siRNAs targeting Cullin-7 (siCul7) to photoreceptors, thereby alleviating photoreceptor ferroptosis by inhibiting Cul7-induced glutathione peroxidase 4 (Gpx4) ubiquitination in N-methyl-N-nitrosourea (MNU)-induced RD mice and Pde6β^rd1/rd1^ mutant mice. Our findings provide a new therapeutic target for photoreceptor loss in RD and establish a PEVs^MH42^ loaded with siCul7 (PEVs^MH42^-siCul7)-based RNAi strategy for RD therapy.

## Materials and Methods

### Ethics

All experiments in this study were approved by the Medical Ethics Committee of Jinhua Central Hospital (approval number: AL-JHYY202429).

### Isolation and identification of PEVs

PEVs were isolated by ultracentrifugation from the cell supernatant of 661W cells cultured with serum-free medium (Life Technologies, USA). Briefly, the conditioned medium was centrifuged at 300 g for 10 min, 2,000 g for 20 min and 10,000 g for 30 min to remove dead cells, cell debris, and organelles. The clarified supernatant was ultracentrifuged at 100,000 g for 90 min to precipitate PEVs. After washing with PBS, PEVs were ultracentrifuged at 100,000 g for 90 min, dissolved with sterile PBS and stored at -80 °C. Transmission electron microscopy (TEM) and NanoSight tracking analysis (NTA) were performed to detect the morphology and size distribution of PEVs. EV-associated markers, including CD9, CD63, Alix, TSG101, and Calnexin, were analyzed by Western blot.

### Conjugation of MH42 peptide to PEVs

The sequence of MH42 peptide was SPALHFLGGGSC. The sequence of MH42-CP05 fusion peptide was SPALHFLGGGSCCRHSQMTVSRL. MH42-CP05 fusion peptides were synthesized via peptide bond without spacer by ChinaPeptides (Shanghai, China). Mass spectrum analysis and ^1^H NMR spectrum analysis were used to characterize the MH42-CP05 fusion peptide. PEVs (5 µg) were incubated with MH42-CP05 fusion peptides (20 µg) on a rotator at 4 °C for 12 h. After washing with PBS, unbound peptides were removed by ultrafiltration with 100 kDa molecular weight cut-off ultrafiltration tubes (Millipore, USA). PEVs^MH42^ were precipitated by ultracentrifugation at 100,000 g for 90 min, and dissolved with sterile PBS.

To determine the binding efficiency, MH42 peptides and MH42-CP05 fusion peptides were labeled with FITC. The FITC fluorescence of the mixture containing FITC-labeled MH42 peptides or MH42-CP05 fusion peptides and PEVs was measured using a fluorescence detection plate reader. After conjugation, unbound peptides were obtained by ultrafiltration. The FITC fluorescence of unbound peptides was measured using a fluorescence detection plate reader and quantified using a calibration curve. The binding efficiency was calculated using the following equation:



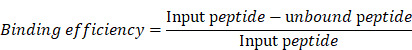



### Cellular uptake of PEVs and PEVs^MH42^

661W cells were seeded at an initial density of 1x10^5^ cells per well in 24-well plates. After incubation for 12 h, the cells were starved in serum-free medium for 12 h. After washing with PBS, several compounds including chlorpromazine (10 μg/mL), indomethacin (10 μg/mL), colchicine (10 μg/mL), and methyl-β-cyclodextrin (10 μg/mL) were added to each well at a volume of 1 mL 16. After the preincubation at 37 °C for 30 min, cells were treated with 1 mL of fresh medium containing 100 µL of PKH26-labeled PEVs (1 × 10^7^ particles/μL) or PEVs^MH42^ (1 × 10^7^ particles/μL). MH42-CP05 fusion peptides were labeled with FITC. After incubation for 48 h, 661W cells were washed with PBS and fixed in 4% paraformaldehyde for 10 min. Following the incubation with DAPI solution (Boster, China) for 10 min at room temperature, cells were observed under a confocal microscope (DeltaVision Elite, GE, USA).

### *In vivo* distribution of PEVs^MH42^

PKH26-labeled PEVs or PEVs^MH42^ were intravitreally injected into the mice. After 24 h, the retinal tissues were isolated, fixed in 4% paraformaldehyde, cryoprotected with 30% sucrose, embedded in the OCT compound, and cut into 15 µm sections. Subsequently, the retinal sections were incubated with rhodopsin antibody (1:200, 27182, CST, USA) or s-opsin antibody (1:200, 27182, CST, USA) overnight. After the incubation with the FITC-conjugated secondary antibodies (Proteintech, China) for 1 h and DAPI solution (Boster, China) for 10 min at room temperature, the sections were photographed using a confocal microscope (DeltaVision Elite, GE, USA).

### Transcriptome sequencing

RNA was extracted from the retinal tissues of normal mice or MNU-induced RD mice and sequenced using an Illumina HiSeq sequencer (Cloundseq, China). Cutadapt, Hisat2 and Cuffdiff software were used to compare high-quality reads to the genome, obtain the FPKM value, and calculate the differentially expressed genes.

### Loading of siRNAs into PEVs^MH42^

Electroporation was used to load siCul7 or siRNA NC into PEVs or PEVs^MH42^. Briefly, 45 μL of PEVs or PEVs^MH42^ (1 × 10^7^ particles/μL) were mixed with 5 μL of siCul7 or siRNA NC (1 μg/μL) in a reaction cup, followed by the electroporation using a CUY21EDIT II electroporator (BEX, Japan) at 110 V and 940 μF (pulse time 15 ms). After electroporation, PEVs loaded with siCul7 (PEVs-siCul7), PEVs^MH42^-siCul7 or PEVs^MH42^ loaded with siRNA NC (PEVs^MH42^-NC) were washed with PBS and exposed to ultracentrifugation at 100,000g for 90 min to remove unbound nucleic acids. To determine loading efficiency, siCul7 and siRNA NC were labeled with FITC. The FITC fluorescence of the mixture containing FITC-labeled siCul7 or siRNA NC and PEVs^MH42^ was measured using a fluorescence detection plate reader. After electroporation, PEVs^MH42^-siCul7 or PEVs^MH42^-NC were precipitated by ultracentrifugation, and unloaded FITC-siCul7 or siRNA NC was obtained from the supernatant. The FITC fluorescence of the supernatant (unloaded siCul7 or siRNA NC) was measured using a fluorescence detection plate reader and quantified using a calibration curve. The loading efficiency was calculated using the following equation:



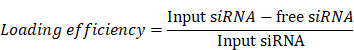



To visualize the PEVs^MH42^-NC and PEVs^MH42^-siCul7, FITC-labeled siCul7 and siRNA NC were introduced into PKH26-labeled PEVs^MH42^ by electroporation, and images were taken using a confocal microscope (DeltaVision Elite, GE, USA). Moreover, the uptake of PEVs^MH42^-NC and PEVs^MH42^-siCul7 by 661W cells and the distribution of PEVs^MH42^-NC and PEVs^MH42^-siCul7 *in vivo* were also detected.

To detect the release of PEVs^MH42^-NC and PEVs^MH42^-siCul7, 1 mL of PEVs^MH42^ loaded with FITC-labeled siRNA NC or siCul7 were added to dialysis bags (100 kDa, Millipore, Germany), which were immersed in 10 mL of PBS (pH 7.4) or PBS (pH 5.0) at 37 °C with orbital shaking at 200 rpm 17. At predetermined time intervals, the samples were withdrawn from the dissolution media and replaced by fresh buffer solution. The released FITC-labeled siRNA NC or siCul7 was quantified using a microplate reader.

### Animal model and treatment

12-week-old C57BL/6 mice were purchased from Cavens Laboratory Animal Center (Changzhou, China) and 2-week-old Pde6β^rd1/rd1^ mutant mice were purchased from Cyagen Bioscience (Suzhou, China). All mice were housed in a pathogen-free environment with a 12 h light/dark cycle at the temperature of 25 °C with free access to food and water. For the establishment of MNU-induced RD mouse model, MNU (Absin, China) was freshly dissolved in sterile saline and injected intraperitoneally into C57BL/6 mice (50 mg/kg). MNU-induced RD mice at 6 h after MNU injection and 2-week-old Pde6β^rd1/rd1^ mice were intravitreally treated with 1 µl of PEVs^MH42^-NC (1 × 10^7^ particles/μL), PEVs^MH42^-siCul7 (1 × 10^7^ particles/μL) or PEVs-siCul7 (1 × 10^7^ particles/μL) with a 33 G Hamilton syringe. All mice at 2 w after PEVs^MH42^-NC, PEVs^MH42^-siCul7 or PEVs-siCul7 treatment were sacrificed with 4% isoflurane and the eyeballs were collected for further analysis. The number of mice used in each experiment is shown in the figure legend.

### Electroretinogram (ERG) analysis

Mice were dark-adapted overnight and intraperitoneally anesthetized with ketamine (87.5 mg/kg) and xylazine (12.5 mg/kg). ERG analysis was performed under dim red light to maintain dark adaptation 18. The corneas were anesthetized with 0.5% proxymetacaine and the pupils were dilated with 0.5% tropicamide. The body temperature was maintained at 37 °C through a heating pad. The ground, reference and record electrodes were placed on the tail, mouth, and cornea, respectively. White flash with an intensity of 3.0 cd.s/m^2^ was used to stimulate the response. ERG recordings were collected using the UTAS Visual Diagnostic System (LKC Technologies, USA).

### Histologic analysis

After enucleation, the retinal tissues were fixed in 4% paraformaldehyde overnight, embedded in paraffin, and sliced into 4 µm sections. After deparaffinization, the retinal sections were stained with hematoxylin and eosin (HE) to observe retinal histology and morphology. The outer nuclear layer (ONL) thickness was quantified using the Image J software along the vertical meridian of the eyeball from nasal to temporal side and through the optic nerve head.

### Immunostaining

After deparaffinization, retinal sections were treated with boiled citrate buffer for antigen retrieval, blocked with 5% bovine serum albumin (BSA) solution for 1 h and incubated with the primary antibody overnight at 4 °C. 661W cells were fixed in 4% paraformaldehyde for 10 min, permeabilized with 0.1% Triton X-100 for 15 min, blocked with 5% BSA solution for 1 h, and incubated with the primary antibody overnight at 4 °C. After incubation with the corresponding fluorescent-conjugated secondary antibodies (Proteintech, China) for 1 h and DAPI solution (Boster, China) for 10 min at room temperature, the sections were observed under a confocal microscope (DeltaVision Elite, GE, USA). The primary antibodies included Cul7 (1:500, sc-514970, Santa Cruz, USA), Ki-67 (1:200, 28074-1-AP, Proteintech, China), rhodopsin (1:200, 27182, CST, USA), s-opsin (1:300, NBP1-20194, Novus, USA), and 4-hydroxynonenal (4-HNE; 1:300, MAB3249, Novus, USA).

### Terminal deoxynucleotidyl transferase dUTP nick end labeling (TUNEL) staining

According to the manufacturer's protocol, TUNEL staining was performed on retinal sections using the TUNEL BrightRed Apoptosis Detection Kit (Vazyme, China). Fixed samples were incubated with 100 µL of proteinase K solution for 20 min and balanced in 100 µL of Equilibration buffer for 30 min, followed by the treatment with 100 µL of TDT buffer for 1 h at 37 °C to label apoptotic cells. After washing with PBS, the retinal sections were counterstained with DAPI solution (Boster, China) and photographed using a confocal microscope (DeltaVision Elite, GE, USA).

### TEM for retinal mitochondrial morphology

After enucleation, retinal tissues were separated and fixed in 2.5% glutaraldehyde phosphate (0.1 M, pH 7.4) overnight at 4 °C. After washing with phosphate buffer, the samples were fixed in osmium acid for 2 h, embedded in EPON, and dehydrated. After cutting and staining with uranyl acetate and lead citrate, the ultrathin sections were observed by TEM (FEI Tecnai 12, Philips, Netherlands).

### Measurement of retinal glutathione (GSH), malondialdehyde (MDA) and iron levels

Retinal GSH, MDA, and iron levels in homogenized retinal tissues were determined using GSH Assay Kit (S0052, Beyotime, China), MDA Assay Kit (S0131S, Beyotime, China) and Iron Assay Kit (ab83366, Abcam, UK) following the manufacturer's instructions, respectively. GSH, MDA, and iron levels were normalized to the retinal protein levels determined using a BCA Assay Kit (P0012S, Beyotime, China).

### Measurement of aqueous humor samples

After disinfection of the conjunctiva, the aqueous humor of MNU-induced RD mouse model and Pde6β^rd1/rd1^ mouse model treated with PEVs^MH42^-NC or PEVs^MH42^-siCul7 was collected using a 33 G Hamilton syringe. Each sample was pooled from the aqueous humor of six eyes from six mice, and five biological repeat samples were quantified for each group. ELISA kits were used to measure the concentrations of cytokines in the samples including IL-1β (SEKM-0002, Solarbio, China), IL-6 (SEKM-0007, Solarbio, China), IL-8 (SEKM-0046, Solarbio, China), and TNF-α (SEKM-0034, Solarbio, China).

### Cell culture and treatment

The cone photoreceptor cell lineage 661W was purchased from the Chinese Academy of Sciences (Shanghai, China). 661W cells were maintained in high-glucose DMEM (Gibco, USA) containing 10% fetal bovine serum, 100 U/ml penicillin, and 100 g/ml streptomycin at 37 °C with 5% CO_2_. *In vitro* treatment of 661W cells with MNU (Absin, China) was conducted at the concentration of 300 µg/ml for 12 h.

### Cell transfection

siCul7 and siRNA NC were designed and synthesized by GenePharma (Suzhou, China). 661W cells were harvested and seeded in 6-well plates (2 × 10^5^ cells/well). When the cell density reached 60%, 661W cells were transfected with siCul7 or siRNA NC using Lipofectamine 2000 (Life Technologies, USA) in serum-free medium according to the manufacturer's instructions. After 6 h, cells were treated with fresh complete medium and cultured for 24 h. The siRNA sequences were as follows:

siRNA NC: sense: UUCUCCGAACGUGUCACGUTT.

siRNA NC: antisense: ACGUGACACGUUCGGAGAATT.

siCul7: sense: GAGCCCAGAACAACUUUAUTT.

siCul7: antisense: AUAAAGUUGUUCUGGGCUCTT.

### Liquid chromatography-tandem mass spectrometry (LC-MS/MS)

50 µg of each protein sample from 661W cells treated with PEVs^MH42^-NC or PEVs^MH42^-siCul7 was subjected to digestion, followed by peptide labeling. The peptides were analyzed by LC-MS/MS. Multiple databases were used for the functional annotation analysis of the identified proteins. Correlation analysis of the differentially expressed proteins was also performed.

### Western blot

The total proteins of retinal tissues, 661W cells, and EVs were isolated using radio-immunoprecipitation assay lysis buffer (Beyotime, China). Equal amounts of protein samples were separated by sodium dodecyl sulfate-polyacrylamide gel electrophoresis and transferred onto polyvinylidene difluoride membranes with a wet-transfer system. Membranes were blocked in 5% skim milk for 1 h and then incubated with primary antibodies overnight at 4 °C. Subsequently, the membranes were washed and incubated with horseradish peroxidase-conjugated secondary antibodies (Proteintech, China) for 2 h at room temperature. The signals were visualized using an ECL detection system (Bio-Rad, USA). Intensity values expressed as the relative protein expression were normalized to β-actin. The primary antibodies included CD9 (1:1000, 98327, CST, USA), CD63 (1:2000, 10112, CST, USA), Alix (1:1000, 92880, CST, USA), TSG101 (1:1000, 72312, CST, USA), Calnexin (1:1000, 10427-2-AP, Proteintech, China), PCNA (1:1000, 10205-2-AP, Proteintech, China), Bcl-2 (1:1000, 26593-1-AP, Proteintech, China), β-actin (1:5000, 4970, CST, USA), Cul7 (1:1000, 67034-1-Ig, Proteintech, China), Gpx4 (1:1000, 67763-1-Ig, Proteintech, China), and Ubiquitin (1:1000, 10201-2-AP, Proteintech, China).

### Co-immunoprecipitation (Co-IP) assay

After treatment, 661W cells were lysed in Co-IP buffer and incubated with the Cul7 antibody (1:100, sc-53810, Santa Cruz, USA) or Gpx4 antibody (1:100, 67763-1-Ig, Proteintech, China) at 4 °C overnight, followed by the incubation with magnetic beads for 4 h. After washing with Co-IP buffer, the protein complexes were detected by western blot.

### Quantitative Real-Time PCR (qRT-PCR)

Total RNA was extracted using TRIzol reagent (Vazyme, China) and reverse transcribed to cDNA using the HiScript II 1st Strand cDNA Synthesis Kit (Vazyme, China) following the manufacturer's protocols. qRT-PCR was performed using the AceQ QPCR SYBR Green Master Mix (Vazyme, China) on an ABI Prism 2720 PCR system. The relative mRNA levels were normalized to β-actin and quantified using the 2^-ΔΔCt^ method. The sequences of primers were as follows:

β-actin: forward: GACCTGTACGCCAACACAGT.

β-actin: reverse: CTCAGGAGGAGCAATGATCT.

Cul7: forward: AGACACGGAAAAGAAGATA.

Cul7: reverse: GCGTCACGACAAGTACACA.

IL-1β: forward: AGCTTCAGGCAGGCAGTATC.

IL-1β: reverse: TCATCTCGGAGCCTGTAGTG.

IL-6: forward: GCTGGAGTCACAGAAGGAGTGGC.

IL-6: reverse: GGCATAACGCACTAGGTTTGCCG.

IL-8: forward: ACTGAGAGTGATTGAGAGTGGAC.

IL-8: reverse: AACCCTCTGCACCCAGTTTTC.

TNF-α: forward: AACTCCAGGCGGTGCCTATG.

TNF-α: reverse: TCCAGCTGCTCCTCCACTTG.

### Cell Counting Kit-8 (CCK8) assay

After treatment, 661W cells were harvested and seeded at an initial density of 2000 cells per well in 96-well plates. After the incubation for 24, 48, 72 and 96 h, cells were treated with 100 µL of fresh medium containing 10 µL of CCK8 reagent (Vazyme, China) for 3 h. The absorbance was measured at 450 nm using an Absorbance Reader (BioTek, USA).

### EdU staining

After transfection, 661W cells were fixed in 4% paraformaldehyde for 30 min and permeabilized with 0.1% Triton X-100 for 15 min. After washing with PBS, cells were stained using the BeyoClick™ EdU-488 Kit (Beyotime, China) and observed under a microscope (Olympus, Japan).

### Statistical analysis

All statistical analysis was performed using the GraphPad Prism software (GraphPad, San Diego, USA). All data were presented as the means ± SEM. Unpaired Student's t-test was used for comparison between two groups. The significant differences among multiple groups were assessed by analysis of variance with Newman-Keuls test. *P* value < 0.05 was considered statistically significant.

## Results

### MH42 efficiently directs PEVs to photoreceptors

**Figure 1:**
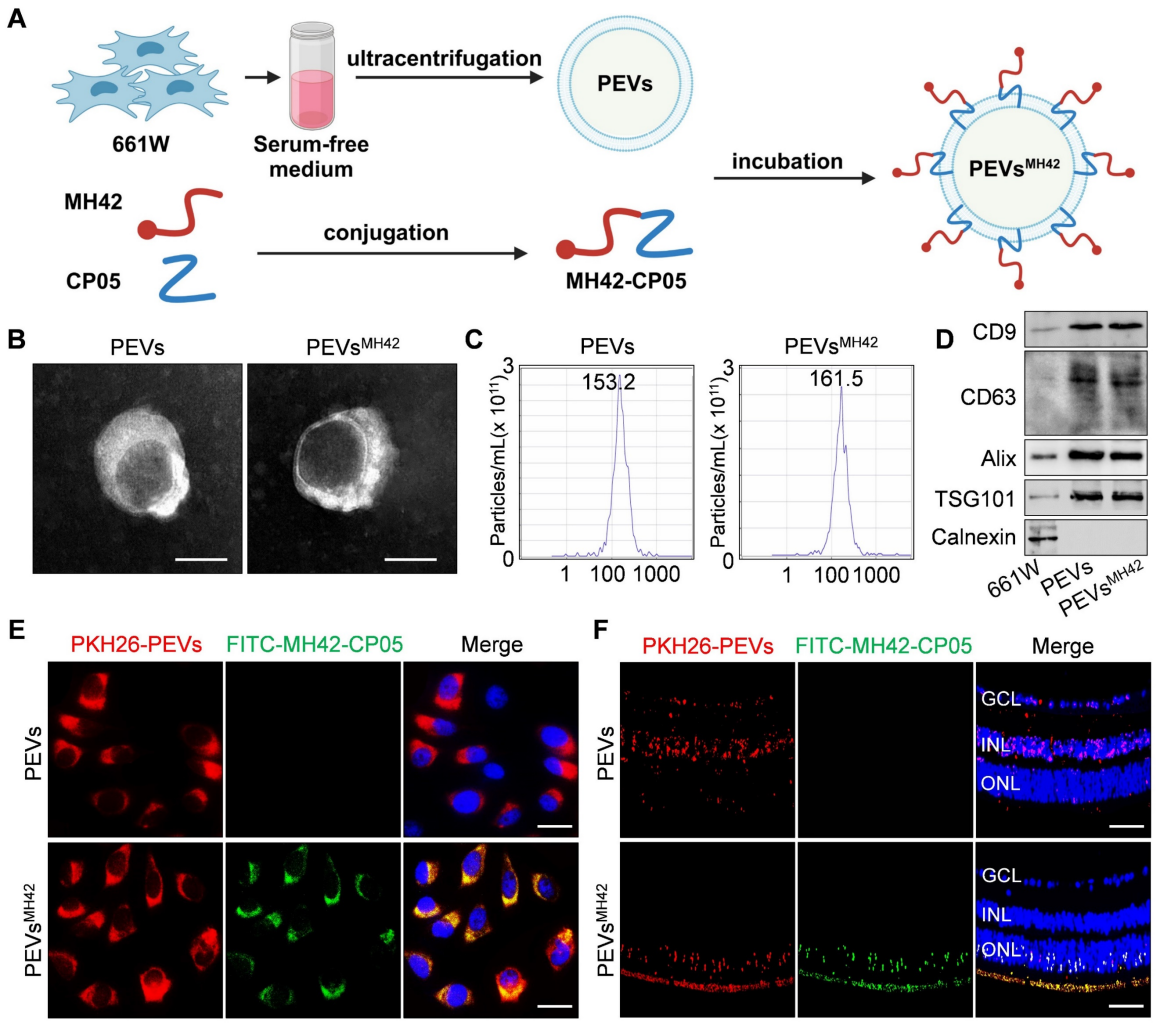
** The preparation and identification of PEVs^MH42^.** (A) Schematic diagram of conjugating MH42 to PEV surface via anchor peptide CP05. (B) Representative TEM images of PEVs and PEVs^MH42^. Scale bars, 100 nm. (C) NTA for the size distribution of PEVs and PEVs^MH42^. (D) Western blot for the protein markers of PEVs and PEVs^MH42^. (E) The internalization of PEVs or PEVs^MH42^ and MH42-CP05 fusion peptide by 661W cells after co-incubation for 48 h. Scale bars, 25 μm. (F) Tracing of FITC-labeled MH42-CP05 fusion peptides and PKH26-labeled PEVs or PEVs^MH42^ in retinal tissues after intravitreal injection for 24 h. Scale bars, 100 μm. GCL, ganglion cell layer; INL, inner nuclear layer; ONL, outer nuclear layer.

To construct the MH42-functionalized PEVs-based delivery system, we isolated PEVs from the serum-free medium of 661W cells and conjugated MH42 onto PEVs using CP05 as an anchor peptide (Figure 1A). Mass spectrum analysis and ^1^H NMR spectrum analysis confirmed the successful synthesis of MH42-CP05 fusion peptide with a molecular weight of 2444 (Figure S1A-B). We observed that MH42-CP05 fusion peptides were specifically located in the photoreceptor-nuclei-residing ONL after intravitreal injection (Figure S1C). TEM images showed that PEVs and PEVs^MH42^ exhibited typical cup-shaped structures (Figure 1B). NTA results revealed that compared with PEVs, there was a slight increase in the diameter of PEVs^MH42^, with the highest peak around 161.5 nm (Figure 1C), indicating that MH42-CP05 fusion peptides were successfully coated onto the surface of PEVs. Western blot demonstrated that PEVs and PEVs^MH42^ expressed EV markers including CD9, CD63, Alix and TSG101, and were negative for the expression of endoplasmic reticulum protein Calnexin (Figure 1D). Based on the FITC-labeled MH42 peptides and MH42-CP05 fusion peptides, we detected their binding efficiency and found that MH42-CP05 fusion peptides could be effectively conjugated with PEVs, whereas MH42 peptides showed low conjugation efficiency (Figure S2).

Subsequently, the delivery efficiency of PEVs^MH42^ was evaluated *in vitro* and *in vivo*. The internalization experiment showed that the modification of MH42 promoted the uptake of PEVs by 661W cells after co-incubation and there was a co-localization between the FITC-labeled MH42-CP05 fusion peptides and PKH26-labeled PEVs (Figure 1E). Endocytosis is recognized as an important pathway for the uptake of EVs 19. Thus, we further investigated the internalization mechanism of PEVs^MH42^ and PEVs by using several endocytosis inhibitors, including chlorpromazine (inhibitor of clathrin-mediated endocytosis), indomethacin (inhibitor of caveolin-mediated endocytosis), colchicine (inhibitor of macropinocytosis), and methyl-β-cyclodextrin (inhibitor of lipid raft-mediated endocytosis) 16. As shown in Figure S3, chlorpromazine pretreatment inhibited the uptake of PEVs^MH42^ and PEVs by 661W cells, whereas other endocytosis inhibitors displayed no significant impact, indicating that the cellular internalization of PEVs^MH42^ and PEVs was mainly through clathrin-mediated endocytosis. Biodistribution analysis also revealed that PEVs were distributed in various retinal layers after intravitreal injection, whereas PEVs^MH42^ and MH42-CP05 fusion peptides were specifically located in the ONL (Figure 1F). Further staining showed that there were co-localizations of PKH26-labeled PEVs^MH42^ with rhodopsin and s-opsin which have been identified as specific cell markers of rods and cones respectively 20, thus verifying the distribution of PEVs^MH42^ in photoreceptors (Figure S4A-B). The above results indicate that MH42 efficiently guides the homing of PEVs to photoreceptors, highlighting the possibility of PEVs^MH42^ as a vector for RD therapy.

### Cul7 is a potential therapeutic target involved in RD pathogenesis

**Figure 2:**
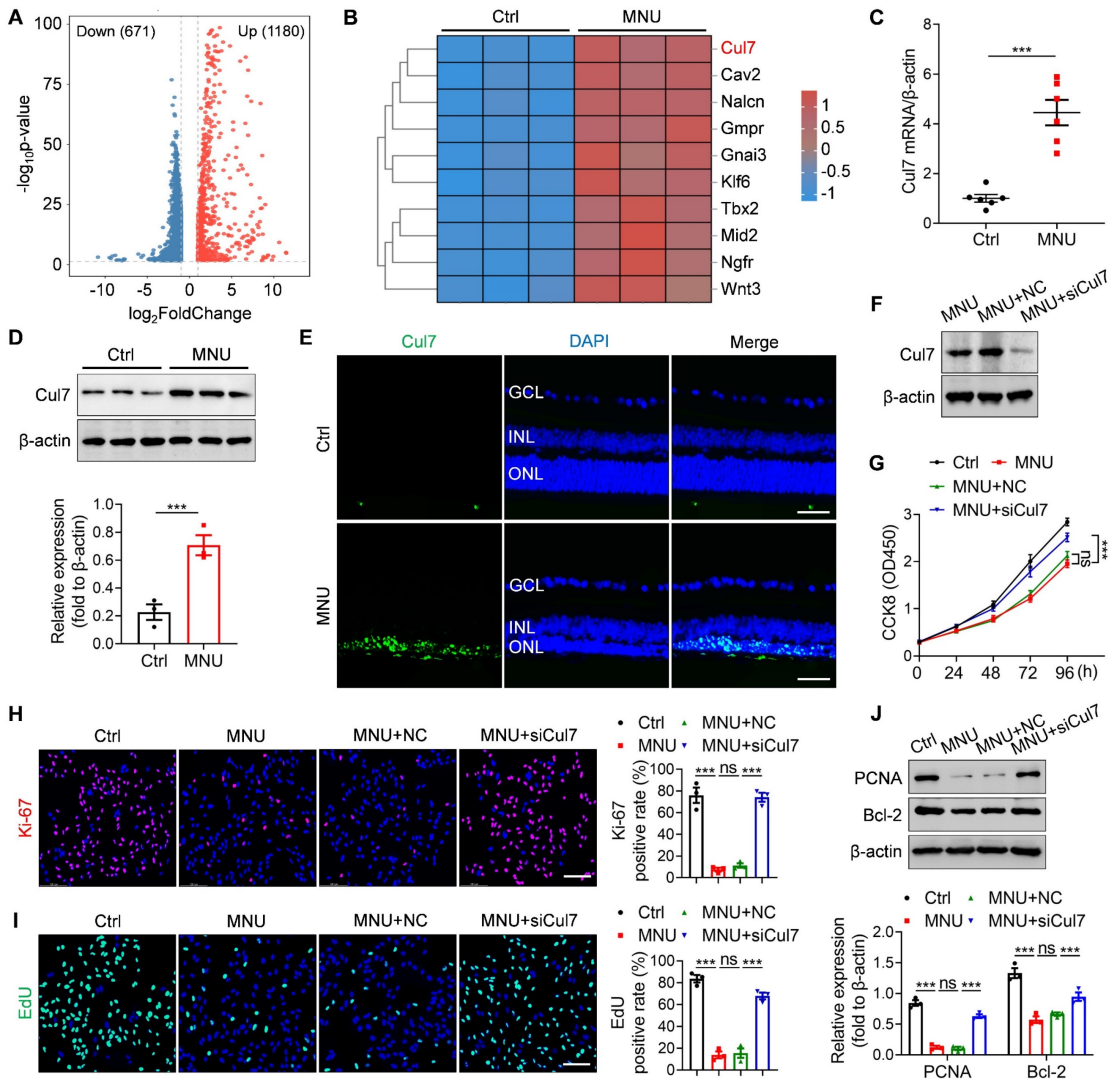
** Cul7 serves as a potential therapeutic target of RD.** (A) Volcano plot for the differentially expressed genes in retinas of normal mice and RD mice by transcriptome sequencing. (B) Heat map of the upregulated genes in retinas of RD mice compared with normal mice. (C) qRT-PCR analysis for the mRNA level of Cul7 in retinal tissues (n=6). (D) Western blot for the expression of Cul7 in retinal tissues. (E) Immunofluorescence staining for the retinal expression of Cul7. Scale bars, 100 μm. (F) Western blot for the expression of Cul7 in 661W cells transfected with siCul7. (G) CCK8 assay for the proliferation of 661W cells transfected with siCul7 (n=4). (H) Immunofluorescence staining for the expression of Ki-67 in 661W cells transfected with siCul7. Scale bars, 100 μm. (I) EdU staining of 661W cells transfected with siCul7. Scale bars, 100 μm. (J) Western blot for the expressions of PCNA and Bcl-2 in 661W cells transfected with siCul7. All data are presented as means ± SEM. ns, not significant, and ***P < 0.001. GCL, ganglion cell layer; INL, inner nuclear layer; ONL, outer nuclear layer.

To identify the potential therapeutic target of RD, we established MNU-induced RD mouse model and performed transcriptome sequencing. As an alkylating agent, MNU can selectively cause photoreceptor death and is commonly used to induce the RD animal model 21, 22. We observed that the intraperitoneal injection of MNU resulted in disordered retinal function, decreased ONL thickness, and significant photoreceptor loss (Figure S5A-D), confirming the successful construction of RD model. Compared with the control group, 1851 genes were differentially expressed in the retinal tissues of MNU-treated mice (Figure 2A). Among them, Cul7 was significantly upregulated (Figure 2B). The elevated Cul7 mRNA level and protein expression in the retinal tissues of MNU-induced RD mice were confirmed by qRT-PCR and Western blot (Figure 2C-D). Immunostaining further showed that MNU treatment promoted the accumulation of Cul7 in the ONL (Figure 2E), indicating that the expression of Cul7 was increased in the photoreceptors during RD progression.

To investigate the role of Cul7 in photoreceptor injury, 661W cells were treated with MNU and then transfected with siCul7. Western blot confirmed that the expression of Cul7 was remarkably downregulated in 661W cells after siCul7 transfection (Figure 2F). Compared with the control group, MNU treatment led to decreased cell proliferation, whereas transfection with siCul7 enhanced the proliferative ability of 661W cells (Figure 2G). The results of Ki-67 immunostaining and EdU staining also showed that Cul7 inhibition enhanced the positive rates of Ki-67 and EdU in 661W cells treated with MNU (Figure 2H-I). Moreover, siCul7 transfection reversed MNU-induced downregulation of PCNA and Bcl-2 in 661W cells (Figure 2J). These findings suggest that Cul7 is an important therapeutic target of RD.

### Targeted inhibition of Cul7 in photoreceptors using siRNA-loaded PEVs^MH42^

**Figure 3:**
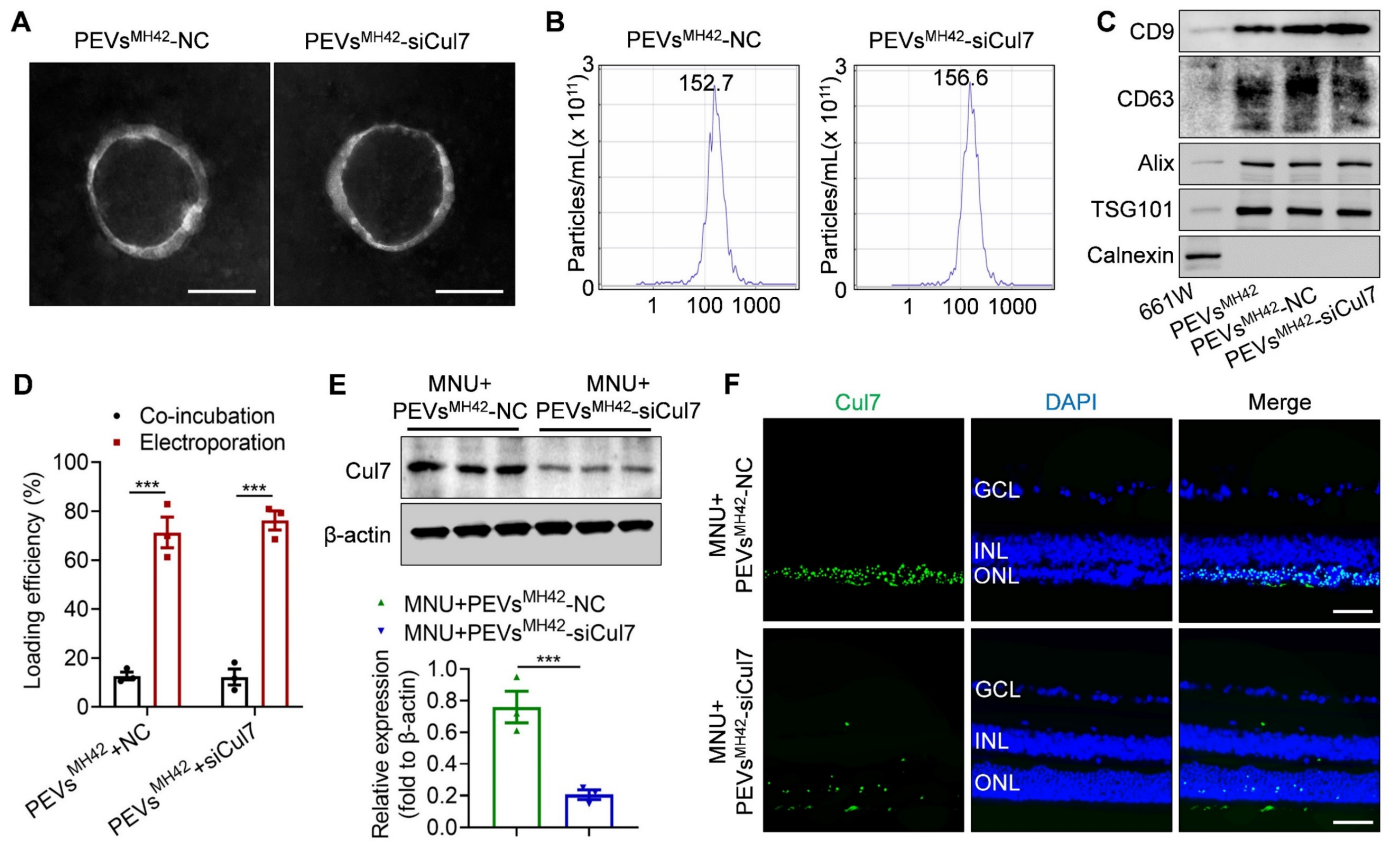
** The preparation of PEVs^MH42^-siCul7.** (A) Representative TEM images of PEVs^MH42^-NC and PEVs^MH42^-siCul7. Scale bars, 100 nm. (B) NTA for the size distribution of PEVs^MH42^-NC and PEVs^MH42^-siCul7. (C) Western blot the protein markers of PEVs^MH42^-NC and PEVs^MH42^-siCul7. (D) The loading efficiency of FITC-labeled siCul7 or siRNA NC by co-incubation or electroporation (n=3). (E) Western blot for the expression of Cul7 in retinal tissues of MNU-induced RD mice treated with PEVs^MH42^-NC or PEVs^MH42^-siCul7. (F) Immunofluorescence staining for the retinal expression of Cul7 in MNU-induced RD mice treated with PEVs^MH42^-NC or PEVs^MH42^-siCul7. Scale bars, 100 μm. All data are presented as means ± SEM. ***P < 0.001. GCL, ganglion cell layer; INL, inner nuclear layer; ONL, outer nuclear layer.

To inhibit Cul7 expression in photoreceptors, we loaded siCul7 into PEVs^MH42^ through electroporation. Using FITC-labeled siRNAs, we quantified the loading efficiency based on the fluorescence intensity and found that the voltage of 110 V and pulse time of 15 ms were suitable electroporation parameters for the introduction of siCul7 and siRNA NC into PEVs^MH42^ (Figure S6). TEM images showed that PEVs^MH42^-NC and PEVs^MH42^-siCul7 maintained the cup-shaped vesicle morphology (Figure 3A and Figure S7A). The average particle diameters of PEVs^MH42^-NC and PEVs^MH42^-siCul7 were 152.7 nm and 156.6 nm respectively (Figure 3B and Figure S7B). The results of Western blot confirmed that PEVs^MH42^-NC and PEVs^MH42^-siCul7 expressed the EV-associated proteins such as CD9, CD63, Alix and TSG101, and negatively expressed Calnexin (Figure 3C). Compared to co-incubation, the loading efficiency of electroporation was significantly improved. Around 71.3% of siRNA NC and 76.2% of siCul7 were introduced into PEVs^MH42^ after electroporation (Figure 3D). The confocal images showed the co-localization of FITC-labeled siRNA NC and siCul7 with PKH26-labeled PEVs^MH42^, further confirming the loading of siRNA NC and siCul7 into PEVs^MH42^ (Figure S8A-B). Moreover, the internalization experiment demonstrated that siRNA NC and siCul7 delivered by PEVs^MH42^ could be uptake by 661W cells (Figure S9A-B). Biodistribution analysis also revealed that PEVs^MH42^ efficiently transmit siRNA NC and siCul7 to the photoreceptors in the ONL (Figure S10A-B). Furthermore, the *in vitro* release profile indicated that PEVs^MH42^-NC and PEVs^MH42^-siCul7 exhibited sustained release characteristics in the conditions with pH 7.4 and 5.0, suggesting that PEVs^MH42^ could reduce siRNA leakage in the physiological environment and protect siRNA from degradation in the intracellular acid environment (Figure S11).

Subsequently, we explored the regulation of PEVs^MH42^-mediated siRNA delivery on Cul7 expression. Western blot demonstrated that the retinal expression of Cul7 was markedly reduced in RD mice treated with PEVs^MH42^-siCul7 compared to the PEVs^MH42^-NC group (Figure 3E). Immunostaining images also showed that PEVs^MH42^-siCul7 prevented the accumulation of Cul7 in the photoreceptors of MNU-induced RD mice (Figure 3F). These results indicate that PEVs^MH42^-siCul7 efficiently deliver siCul7 to the injured photoreceptors, leading to the robust inhibition of Cul7.

### PEVs^MH42^-siCul7 alleviate MNU-induced RD

**Figure 4:**
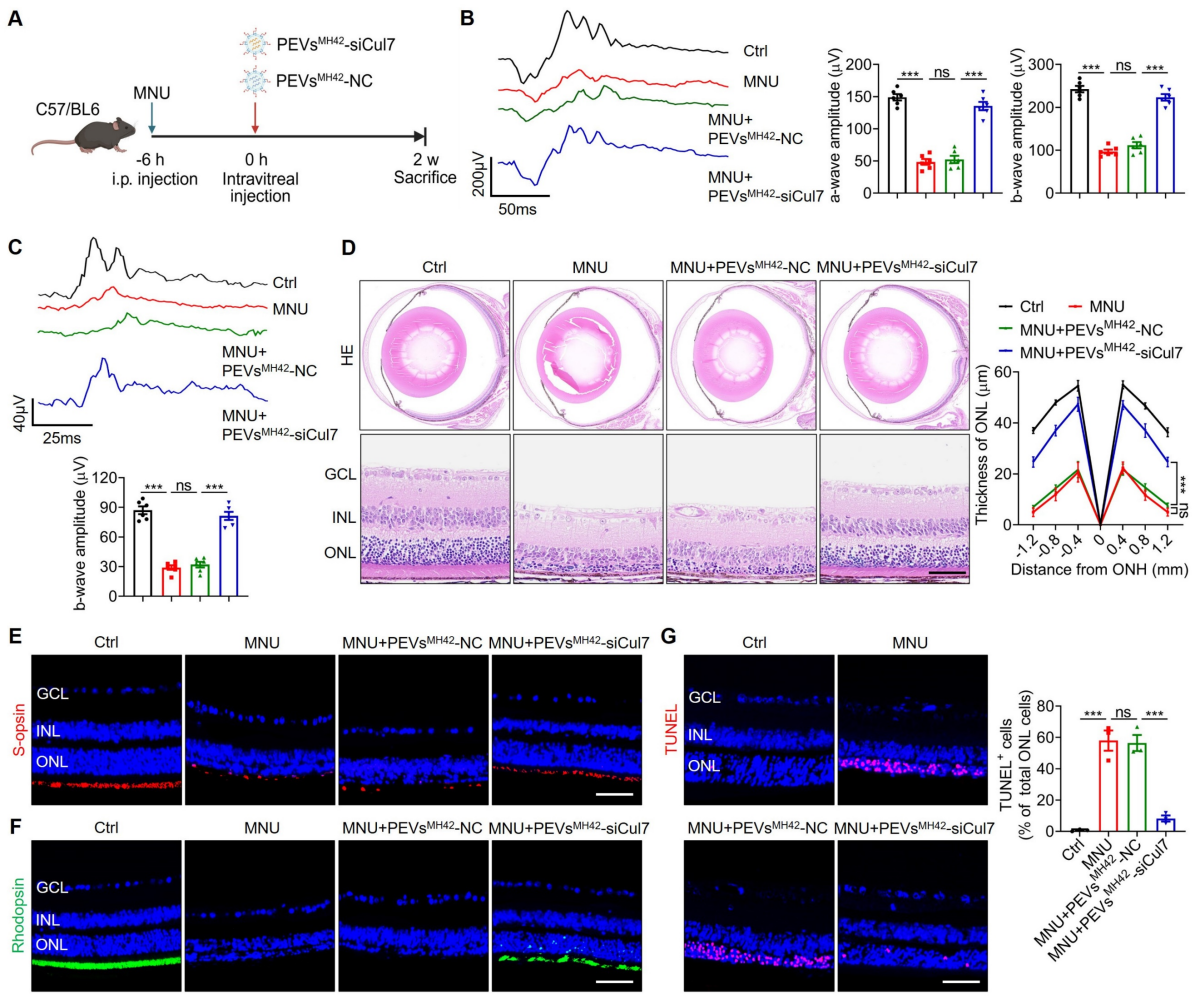
** Therapeutic efficacy of PEVs^MH42^-siCul7 in MNU-induced RD mice.** (A) Schematic diagram showing the study design of MNU-induced RD mouse model. (B) Representative scotopic ERG waveforms and the corresponding quantitative analysis of scotopic a-wave and b-wave amplitudes (n=6). (C) Representative photopic ERG waveforms and the corresponding quantitative analysis of photopic b-wave amplitude (n=6). (D) HE staining of retinal tissues and the corresponding quantitative analysis of ONL thickness (n=3). Scale bars, 100 μm. (E) Immunofluorescence staining for the retinal expression of s-opsin. Scale bars, 100 μm. (F) Immunofluorescence staining for the retinal expression of rhodopsin. Scale bars, 100 μm. (G) TUNEL staining of retinal tissues and the corresponding quantitative analysis of TUNEL^+^ cell percentage of total ONL cells (n=3). Scale bars, 100 μm. All data are presented as means ± SEM. ns, not significant, and ***P < 0.001. GCL, ganglion cell layer; INL, inner nuclear layer; ONL, outer nuclear layer.

We then evaluated the therapeutic efficacy of PEVs^MH42^-siCul7 in RD. The phototoxin MNU was used to establish the photoreceptor-specific injury model in mice, followed by the intravitreal injection of PEVs^MH42^-siCul7 or PEVs^MH42^-NC (Figure 4A). We examined retinal electrophysiological function by ERG analysis including scotopic and photopic responses, which reflect the hyperpolarization of photoreceptors and the subsequent depolarization of bipolar cells postsynaptic to photoreceptors 23. As detected, intraperitoneal injection of MNU caused injured retinal function, quantified by the decreased amplitudes of dark-adapted scotopic a/b-waves as well as the light-adapted photopic b-wave. Compared to PEVs^MH42^-NC, PEVs^MH42^-siCul7 protected the photoreceptor activity of MNU-induced RD mice (Figure 4B-C). HE staining of retinal tissues showed that PEVs^MH42^-siCul7 treatment ameliorated MNU-triggered retinal degeneration and improved ONL thickness, whereas PEVs^MH42^-NC exerted limited effects (Figure 4D). Immunostaining revealed the low survival of s-opsin^+^ cones and rhodopsin^+^ rods in mice with RD, while PEVs^MH42^-siCul7 alleviated the loss of cone and rod photoreceptors (Figure 4E-F). TUNEL staining also demonstrated that relative to PEVs^MH42^-NC, PEVs^MH42^-siCul7 effectively inhibited photoreceptor apoptosis in the retinal ONL of MNU-treated mice (Figure 4G). Moreover, ocular inflammation, a common complication of intravitreal injection, was evaluated in MNU-induced RD mice after intravitreal treatment with PEVs^MH42^-siCul7 or PEVs^MH42^-NC. We observed that PEVs^MH42^-siCul7 or PEVs^MH42^-NC intervention did not significantly affect the levels of inflammatory cytokines including IL-1β, IL-6, IL-8 and TNF-α in the aqueous humor samples and retinal tissues (Figure S12A-B), confirming the safety of PEVs^MH42^-siCul7-mediated treatment strategy. These data indicate that PEVs^MH42^-siCul7 treatment can counteract MNU-induced photoreceptor injury and alleviate RD progression.

### PEVs^MH42^-siCul7 attenuate RD in Pde6β^rd1/rd1^ mutant mice

**Figure 5:**
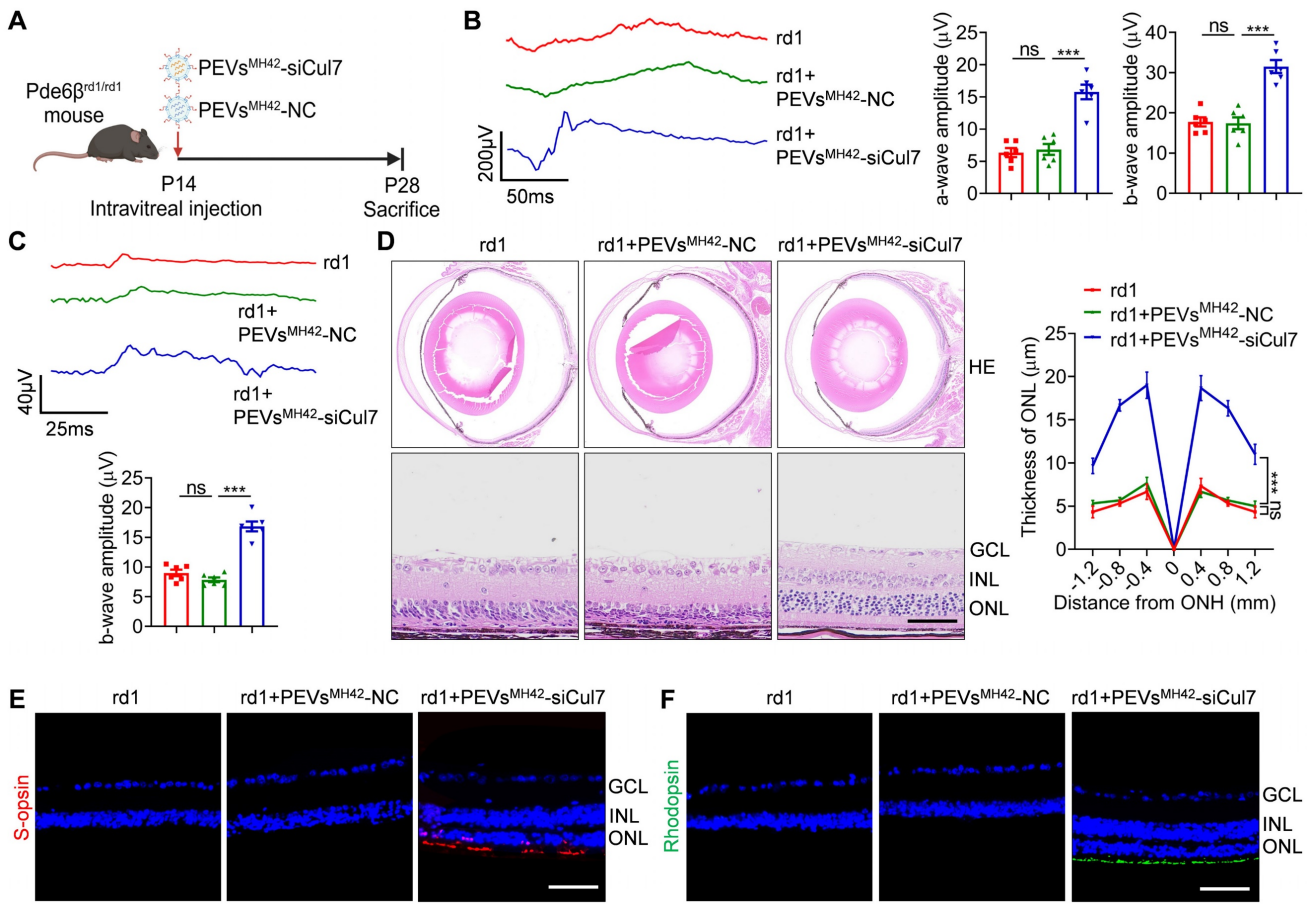
** Therapeutic role of PEVs^MH42^-siCul7 in Pde6β^rd1/rd1^ mutant mice.** (A) Schematic diagram showing the study design of Pde6β^rd1/rd1^ mutant mice. (B) Representative scotopic ERG waveforms and the corresponding quantitative analysis of scotopic a-wave and b-wave amplitudes (n=6). (C) Representative photopic ERG waveforms and the corresponding quantitative analysis of photopic b-wave amplitude (n=6). (D) HE staining of retinal tissues and the corresponding quantitative analysis of ONL thickness (n=3). Scale bars, 100 μm. (E) Immunofluorescence staining for the retinal expression of s-opsin. Scale bars, 100 μm. (F) Immunofluorescence staining for the retinal expression of rhodopsin. Scale bars, 100 μm. All data are presented as means ± SEM. ns, not significant, and ***P < 0.001. GCL, ganglion cell layer; INL, inner nuclear layer; ONL, outer nuclear layer.

To further confirm whether PEVs^MH42^-siCul7 serve as a promising strategy for RD therapy, we used Pde6β^rd1/rd1^ mutant mice which is a genetic mouse RD model with the same mutation of Pde6β as retinitis pigmentosa patients 24. According to the previous study showing that Pde6β^rd1/rd1^ mutant mouse model exhibits rapid RD 25, we treated Pde6β^rd1/rd1^ mutant mice with PEVs^MH42^-siCul7 or PEVs^MH42^-NC intravitreally at P14 (the early stage of photoreceptor degeneration), and evaluate their retinal therapeutic effects at P28 (Figure 5A). The results of both scotopic and photopic analysis demonstrated that the application of PEVs^MH42^-siCul7 resulted in the increased amplitudes of scotopic a/b-wave and photopic b-wave, indicating that PEVs^MH42^-siCul7 could preserve the retinal function of Pde6β^rd1/rd1^ mutant mice (Figure 5B-C). Histologic analysis of the retinas from Pde6β^rd1/rd1^ mutant mice showed the disturbed structure with dramatically reduced ONL thickness. Compared with PEVs^MH42^-NC intervention, PEVs^MH42^-siCul7-treated Pde6β^rd1/rd1^ mutant mice presented a recovered morphology with increased ONL thickness (Figure 5D). Moreover, immunostaining revealed that the photoreceptors of Pde6β^rd1/rd1^ mutant mice could be protected by PEVs^MH42^-siCul7, whereas PEVs^MH42^-NC provided limited therapeutic effects (Figure 5E-F). In addition, ELISA and qRT-PCR results demonstrated that the levels of inflammatory cytokines in the aqueous humor samples and retinal tissues of Pde6β^rd1/rd1^ mutant mice after PEVs^MH42^-siCul7 or PEVs^MH42^-NC injection were not remarkably altered, indicating that the intravitreal delivery of PEVs^MH42^-siCul7 or PEVs^MH42^-NC did not cause ocular inflammation (Figure S13A-B). Therefore, the above results suggest that PEVs^MH42^-siCul7 can effectively protect photoreceptors to alleviate RD both in MNU-induced RD mice and Pde6β^rd1/rd1^ mutant mice.

Furthermore, we investigated the role of MH42 peptide in the targeting potential and retinal therapeutic value of PEVs^MH42^-siCul7. Biodistribution analysis showed that PEVs-siCul7 were distributed in various retinal layers and rarely located in the photoreceptor-nuclei-residing ONL after intravitreal injection, while PEVs^MH42^-siCul7 were mainly distributed in the ONL (Figure S14A). Immunostaining revealed that PEVs-siCul7 exerted limited effects to inhibit Cul7 expression in photoreceptors (Figure S14B). Moreover, compared with PEVs^MH42^-siCul7 group, the therapeutic efficiency of PEVs-siCul7 to improve retinal function and alleviate photoreceptor loss was significantly decreased both in MNU-induced RD mice and Pde6β^rd1/rd1^ mutant mice (Figure S14C-J), indicating that MH42 peptide modification is essential for the ability of PEVs^MH42^-siCul7 to target photoreceptors and ameliorate RD progression.

### PEVs^MH42^-siCul7 inhibit Cul7-mediated Gpx4 ubiquitination

**Figure 6:**
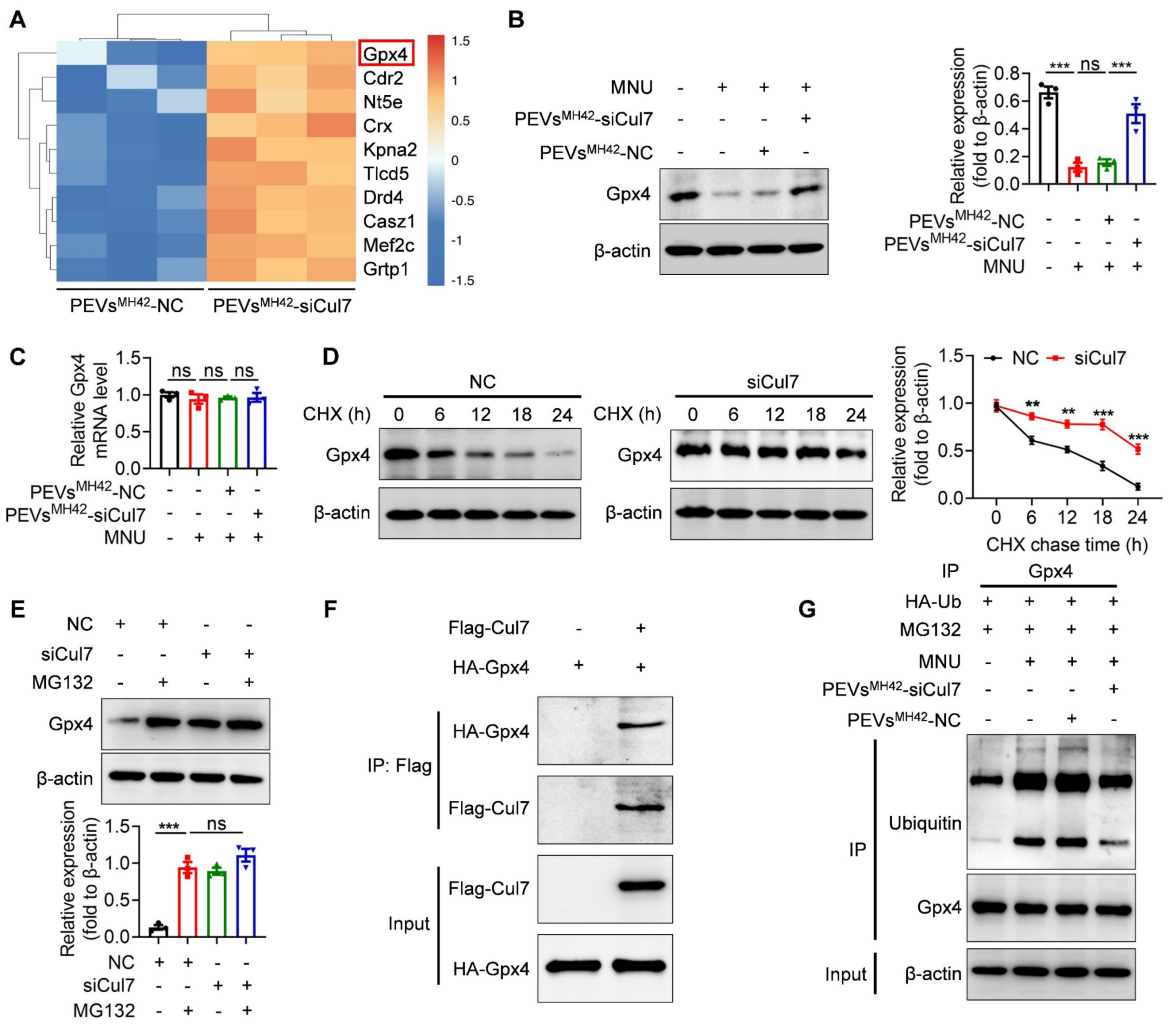
** PEVs^MH42^-siCul7 inhibit Cul7-mediated Gpx4 ubiquitination and degradation.** (A) LC-MS/MS analysis for the differentially expressed proteins in 661W cells treated with PEVs^MH42^-NC or PEVs^MH42^-siCul7. (B) Western blot for the protein expression of Gpx4 in 661W cells. (C) qRT-PCR for the mRNA level of Gpx4 in 661W cells (n=3). (D) CHX assay for the half-time of Gpx4 protein. (E) The effect of MG132 on Gpx4 expression. (F) Co-IP assay for the binding of Cul7 protein to Gpx4 protein. (G) Co-IP assay for the ubiquitination level of Gpx4 in 661W cells treated with PEVs^MH42^-NC or PEVs^MH42^-siCul7. All data are presented as means ± SEM. ns, not significant, **P < 0.01, and ***P < 0.001.

Subsequently, we intended to explore the molecular mechanism underlying the retinal therapeutic effects of PEVs^MH42^-siCul7. LC-MS/MS analysis showed the upregulation of Gpx4 in 661W cells after PEVs^MH42^-siCul7 treatment (Figure 6A). Western blot and qRT-PCR confirmed that PEVs^MH42^-siCul7 could promote the protein expression of Gpx4, whereas Gpx4 mRNA level was not significantly altered in 661W cells treated with PEVs^MH42^-siCul7 (Figure 6B-C), implying that PEVs^MH42^-siCul7-mediated Cul7 inhibition may regulate the stability of Gpx4 protein. To substantiate this assumption, we transfected 661W cells with siCul7 and detected the half-life of Gpx4 protein after protein synthesis inhibitor cycloheximide (CHX) treatment. Compared to the control group, the half-life of Gpx4 protein in the siCul7 transfection group was extended (Figure 6D). Moreover, the proteasome inhibitor MG132 was observed to reverse the Cul7-induced reduction of Gpx4 protein level (Figure 6E), indicating that Cul7 could regulate Gpx4 protein stability mainly through the ubiquitin-proteasome pathway. As an E3 ubiquitin ligase, Cul7 participate in the ubiquitination modification of protein 26. The results of Co-IP assay further demonstrated that Cul7 protein could bind to Gpx4 protein and PEVs^MH42^-siCul7 prevented Cul7-mediated Gpx4 ubiquitination and degradation (Figure 6F-G).

### PEVs^MH42^-siCul7 prevent photoreceptor ferroptosis

**Figure 7:**
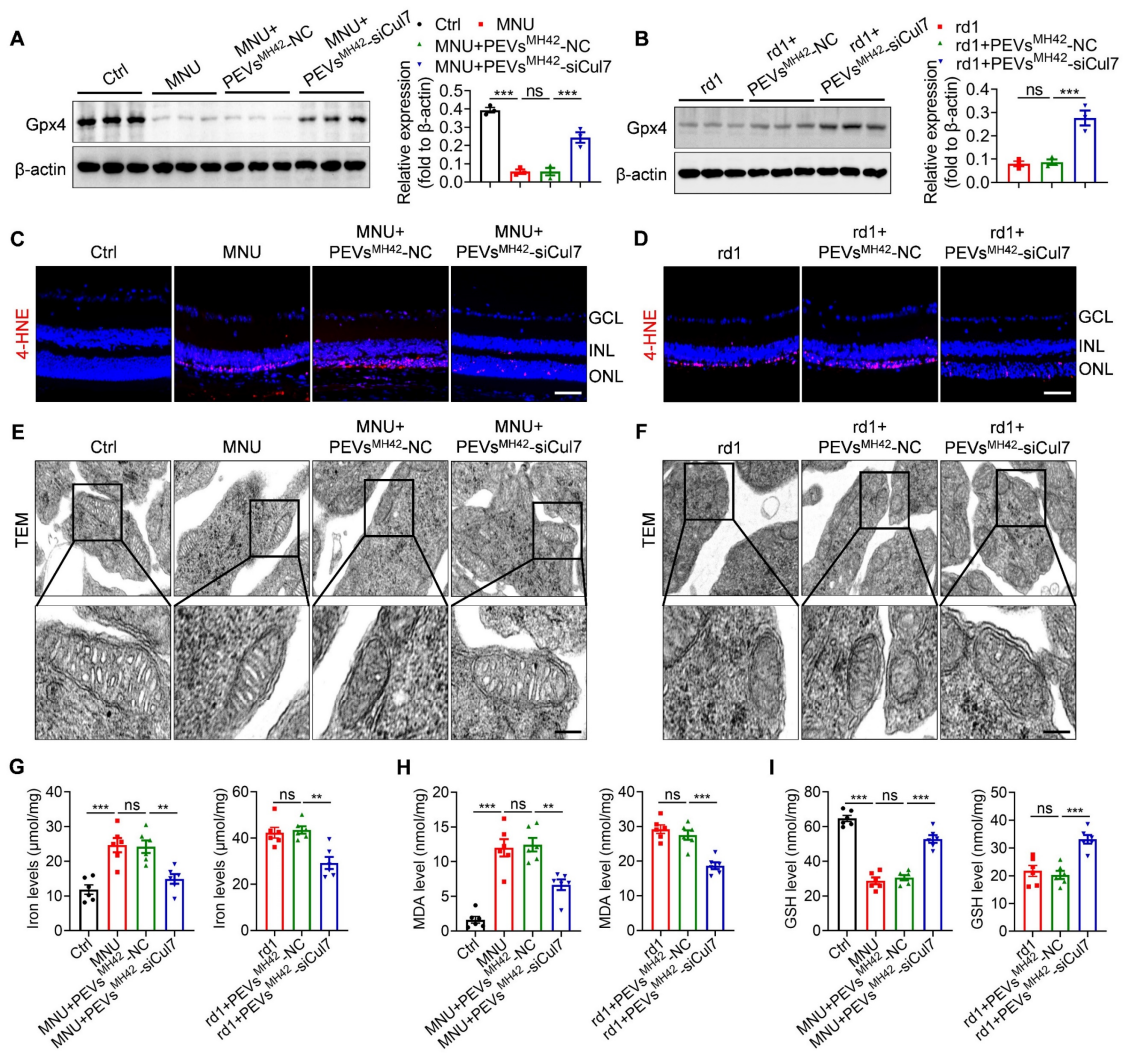
** PEVs^MH42^-siCul7 inhibit photoreceptor ferroptosis in MNU-induced RD mice and Pde6β^rd1/rd1^ mutant mice.** (A, B) Western blot for the retinal expression of Gpx4 in MNU-induced RD mice and Pde6β^rd1/rd1^ mutant mice. (C, D) Immunofluorescence staining for the retinal expression of 4-HNE in MNU-induced RD mice and Pde6β^rd1/rd1^ mutant mice. Scale bars, 100 μm. (E, F) TEM images of mitochondria in photoreceptors of MNU-induced RD mice and Pde6β^rd1/rd1^ mutant mice. Scale bars, 200 nm. (G-I) Measurement of iron, MDA and GSH levels in retinal tissues of MNU-induced RD mice and Pde6β^rd1/rd1^ mutant mice (n=6). All data are presented as means ± SEM. ns, not significant, **P < 0.01, and ***P < 0.001. GCL, ganglion cell layer; INL, inner nuclear layer; ONL, outer nuclear layer.

By catalyzing the reduction of phospholipid peroxides, Gpx4 is reported to act as an important inhibitor of ferroptosis 27. Thus, we explored whether PEVs^MH42^-siCul7-induced Gpx4 activation could regulate photoreceptor ferroptosis, resulting in the alleviation of RD. Western blot results showed that MNU injection significantly reduced retinal Gpx4 expression, whereas PEVs^MH42^-siCul7 treatment reversed MNU-induced Gpx4 downregulation in retinal tissues (Figure 7A). Similarly, intravitreal injection of PEVs^MH42^-siCul7 also enhanced the Gpx4 protein level in retinas of Pde6β^rd1/rd1^ mutant mice (Figure 7B). Immunostaining images revealed that the toxic lipid peroxidation product 4-HNE accumulated in the retinal ONL of MNU-induced RD mice and Pde6β^rd1/rd1^ mutant mice, which could be prevented by PEVs^MH42^-siCul7 treatment (Figure 7C-D). Subsequently, we observed the ultrastructure of mitochondria in the photoreceptors using TEM. As shown in Figure 7E-F, the mitochondrial morphology of photoreceptors from MNU-treated mice and Pde6β^rd1/rd1^ mutant mice exhibited increased mitochondrial membrane density with reduced mitochondrial crista structures, which is consistent with a typical feature previously reported during ferroptosis 28. After PEVs^MH42^-siCul7 intervention, the structure of mitochondria was restored. Moreover, the ferroptosis-related indicators, including iron level, lipid peroxide MDA and antioxidant GSH, were determined. We found that PEVs^MH42^-siCul7 could reduce the levels of iron and MDA, and enhance the GSH level in retinas of MNU-induced RD mice and Pde6β^rd1/rd1^ mutant mice (Figure 7G-I). Together, these findings demonstrate that photoreceptor ferroptosis is a major pathological process involved in RD and PEVs^MH42^-siCul7 effectively protect photoreceptors against ferroptosis.

## Discussion

RNAi-mediated gene therapy has emerged as a precise and personalized strategy for various refractory diseases 29. For RD therapy, photoreceptor-targeted transfer of RNAi therapeutics and lack of effective therapeutic targets are the main concerns. In this study, we prepared a novel PEVs-based delivery platform modified with the photoreceptor-binding peptide MH42 on the surface and internally loaded with siCul7 to alleviate RD. PEVs^MH42^-siCul7 were observed to efficiently deliver siCul7 to photoreceptors and prevent photoreceptor ferroptosis by inhibiting Cul7-mediated Gpx4 ubiquitination and degradation.

Increasing studies have demonstrated that siRNA-based gene silencing is a promising strategy for the treatment of various diseases 30. However, the delivery of siRNAs has several limitations including poor stability, safety and cellular uptake 31. Recently, the use of EVs as nanocarriers for siRNA delivery has attracted widespread attention due to their favorable properties such as low toxicity and immunogenicity, high stability, intrinsic ability to cross biological barriers and cargo protection capacity 32. Nevertheless, Mathew *et al.* have shown that EVs do not possess the ability to target specific retinal cells after intravitreal injection, resulting in the reduced therapeutic efficiency 33. The accumulation of EVs in non-specific organs is a major challenge in EV-based delivery systems, leading to insufficient siRNA delivery to targeted tissues or cells 34. Recent evidence has suggested that nanoparticles can be modified to enhance their retinal permeability and therapeutic efficacy in ocular diseases 35. Li *et al.* have developed Asp-Gly-Arg peptide-modified solid lipid nanoparticles to efficiently deliver miR-150 and quercetin to endothelial cells for age-related macular degeneration therapy 36. Accumulating studies have revealed that the targeting potential of EVs can also be improved through surface modification with targeting peptides 37. The engineering of donor cells to release targeting peptide-conjugated EVs is a common method. However, this strategy is complex, time-consuming, and cannot be applied to pre-isolated EVs 38. Therefore, it is necessary to develop biochemical methods to modify EVs directly. Recently, Gao *et al.* have demonstrated the specific binding between the CP05 anchor peptide and CD63, a tetraspanin enriched on the surface of EVs 39. Zha *et al.* have combined 3D-printed porous bone scaffolds with EVs via CP05 anchor peptides to increase osteogenesis and angiogenesis in segmental bone defects 40. However, the application of CP05 peptides for the surface functionalization of EVs to achieve the targeted delivery of siRNA has not yet been reported. MH42 is a newly identified peptide that is specifically taken up by photoreceptors 15. Conjugation of MH42 may promote the accumulation of EVs in photoreceptors. In this study, considering the natural homing property of EVs, we chose 661W cell-derived EVs as the siRNA carriers. Based on the CP05 peptide-mediated interaction with CD63, we constructed MH42-CP05 fusion peptides and conjugated MH42 onto EV membranes by co-incubation. PEVs^MH42^ were found to be mainly distributed in photoreceptors after intravitreal injection, thereby providing a foundation to efficiently deliver siRNA to photoreceptors for the targeted treatment of RD.

Another major challenge associated with RD therapy is the lack of effective therapeutic targets. Photoreceptor loss caused by multiple factors is considered a major pathological mechanism underlying RD 41. Recent studies have indicated that the ubiquitin-proteasome system exerts an important role in the functional regulation of photoreceptors during RD progression 42, 43. Here, we performed transcriptome sequencing to explore the critical genes in inducing photoreceptor injury. Notably, a significant upregulation of Cul7 was observed in the photoreceptors of MNU-induced RD mice. As an E3 ubiquitin ligase, Cul7 contributes to the post-translational modification of cellular proteins 44. Current studies mainly focus on the function of Cul7 in cancer. The overexpression of Cul7 can promote the tumorigenesis of glioma 45. Cul7-mediated Caspase-8 ubiquitination enables breast cancer cells with anti-apoptotic roles 46. Cul7 also shows the potential as a promising prognostic marker for colon adenocarcinoma 47. However, the association between Cul7 activation and photoreceptor damage in RD is still unclear. In this study, we transfected 661W cells with siCul7 and found that Cul7 knockdown promoted the survival of MNU-treated photoreceptors, suggesting that Cul7 inhibition contributes to the alleviation of photoreceptor injury in RD.

In the treatment of retinal diseases, the movement of nanoparticles through the vitreous is influenced by their size relative to the vitreous pore size. Xu *et al.* have shown that nanoparticles with a diameter of less than 500 nm can diffuse rapidly through the meshwork pores of vitreous, while macromolecules are restricted by the barriers owing to the average vitreous mesh pore size of 550 nm 48. Although several nanoparticles, such as nanoemulsions, nanomicelles, quantum dots, liposomes, and inorganic nanoparticles, show ocular drug delivery potential, certain toxicities have been detected in the cornea, conjunctiva, and retina 49. Compared to these nanoparticles, EVs seem to be more suitable for ophthalmological applications because of their favorable safety profiles and few side effects 50. Moreover, cell-penetrating peptides have been reported to cross the membrane and transport membrane-impermeable cargos in the eye 51. However, despite the encouraging results, cell-penetrating peptides show off-target effects due to their free diffusion and they are still characterized by instability *in vivo* because of their susceptibility to ubiquitous proteases 52. Compared to cell-penetrating peptides, EVs represent a valuable alternative in virtue of their stability in the circulation and enhanced cell-specific targeting after modification 53. In this study, we loaded siRNA NC and siCul7 into PEVs^MH42^ through electroporation and found that PEVs^MH42^-NC and PEVs^MH42^-siCul7 maintained the characteristics of PEVs with the average particle diameter of 152.7 nm and 156.6 nm respectively. As expected, PEVs^MH42^-NC and PEVs^MH42^-siCul7 readily migrated through the vitreous and effectively delivered siRNA NC and siCul7 to photoreceptors based on the PEVs^MH42^-mediated targeted delivery platform.

Gene therapy is a promising strategy for the treatment of ocular diseases including RD. The efficient uptake of genetic drugs by target cells is essential for their specific activity. The particular conditions governing the safety and feasibility of gene therapy are dictated by the choice of delivery tool 54. The Adeno-associated virus (AAV) is a widely used vector for gene therapy because of its excellent safety profile and high gene transduction efficiency 55. Karali *et al.* have shown that AAV vector carrying the miR-204 precursor intervention exerts a potent role to attenuate microglia activation and photoreceptor cell death in RD 56. The results of Sun *et al.* have demonstrated that photoreceptor-specific AAV vector-mediated delivery of shRNA can inhibit c-Fos expression to alleviate pathological neovascularization and rescue visual function 57. However, ocular delivery of clinical grade AAV can cause systemic immune responses and ocular inflammation 58. The packaging capacity of AAV is also low and limited to 4.5 kb 59. In addition, synthetic nanoparticles have recently been used as carriers for gene therapy due to their nanoscale size. For instance, the self-assembled nanoparticles-induced specific and prolonged ABCA4 gene expression in the outer segment of photoreceptors contributes to the amelioration of visual dystrophies 60. Nevertheless, the efficiency of synthetic nanoparticles is lower than that of AAV vectors 61. The formulation of many synthetic nanoparticles is difficult because some compounds are highly viscous, which may prevent their wide distribution in retinal tissues 62. In this study, we demonstrated that employing PEVs^MH42^ to deliver siCul7 effectively downregulated Cul7 expression in photoreceptors, thereby attenuating retinal dysfunction, structural degeneration, and photoreceptor loss both in MNU-induced RD mice and Pde6β^rd1/rd1^ mutant mice. Furthermore, PEVs^MH42^-siCul7 treatment did not cause inflammatory responses, confirming their biosafety in RD therapy.

Ferroptosis is an iron-dependent form of cell death caused by the redox imbalance between the production of oxidants and antioxidants 63, 64. Increasing evidence has suggested that ferroptosis is closely associated with RD. Deleon *et al.* have demonstrated that the levels of ferroptosis-related markers, including ferritin and 4-HNE, are significantly increased in rd10 mice 65. Moreover, the intravitreal injection of Fe^2+^ can cause photoreceptor oxidative stress and degeneration, while upregulating 4-HNE level and downregulating Gpx4 level 66. In the present study, LC-MS/MS analysis revealed that PEVs^MH42^-siCul7-mediated Cul7 inhibition upregulated Gpx4 protein level in 661W cells. As an important antioxidant enzyme, Gpx4 prevents ferroptosis by converting lipid hydroperoxides into non-toxic lipid alcohols 67. The inhibition of ferroptosis by the induction of Gpx4 is considered a therapeutic strategy for many diseases 68. However, the role of Cul7 in ferroptosis regulation and the interaction between Cul7 and Gpx4 remain unclear. Herein, our findings indicated that Cul7 promoted Gpx4 ubiquitination and degradation by directly binding to Gpx4. PEVs^MH42^-siCul7-induced Gpx4 activation could remarkably inhibited photoreceptor ferroptosis, thus preventing photoreceptor loss in RD.

However, it is important to note that the rodent models used to study RD have certain limitations in replicating disease progression of human patients. Therefore, studies involving nonhuman primate models and human patients with RD should be considered for determining the dosage, injection timing and biosafety of PEVs^MH42^-siCul7. Further explorations are required to investigate the clinical applicability of PEVs^MH42^-siCul7 in RD therapy.

## Conclusions

In summary, we have developed a photoreceptor-targeted delivery platform and highlighted its potential in the transfer of siCul7 as a promising therapy for RD. Our results indicated that PEVs^MH42^-siCul7 modified with the photoreceptor-targeted peptide MH42 efficiently homed to and delivered siCul7 to photoreceptors. Consequently, suppression of Cul7 inhibited Cul7-mediated Gpx4 ubiquitination and degradation and upregulated Gpx4 expression, thus preventing photoreceptor ferroptosis in MNU-induced RD mice and Pde6β^rd1/rd1^ mutant mice. Our study suggests the transformative potential of PEVs^MH42^-siCul7 in protecting photoreceptors and supplies a unique platform that may accelerate the development of gene therapy for RD.

## Supplementary Material

Supplementary figures and tables.

## Abbreviations

BSA: bovine serum albumin
CCK8: Cell Counting Kit-8
CHX: cycloheximide
Co-IP: co-immunoprecipitation
Cul7: Cullin-7
ERG: electroretinogram
EVs: extracellular vesicles
Gpx4: glutathione peroxidase 4
GSH: glutathione
HE: hematoxylin and eosin
LC-MS/MS: liquid chromatography-tandem mass spectrometry
MDA: malondialdehyde
MNU: N-methyl-N-nitrosourea
NTA: nanoSight tracking analysis
ONL: outer nuclear layer
PEVs: photoreceptor-derived EVs
PEVs^MH42^: PEVs conjugated with MH42 peptide
PEVs^MH42^-siCul7: PEVs^MH42^ loaded with siCul7
qRT-PCR: quantitative Real-Time PCR
RD: retinal degeneration
RNAi: RNA interference
siCul7: siRNAs targeting Cul7
siRNA: small-interfering RNA
TEM: transmission electron microscopy
TUNEL: terminal deoxynucleotidyl transferase dUTP nick end labelling
4-HNE: 4-hydroxynonenal
