# Supplementary material for: Photoreceptor-targeted extracellular vesicles-mediated delivery of Cul7 siRNA for retinal degeneration therapy
Source: Theranostics. 2024 Aug 12;14(13):4916–32. doi: 10.7150/thno.99484 (PMC11388070; doi:10.7150/thno.99484)
Supplement: Supplementary file 1 — Supplementary figures and tables. [file thnov14p4916s1.pdf]

Supplementary Materials for

Photoreceptor-targeted extracellular vesicles-mediated delivery of Cul7 siRNA for retinal degeneration therapy

Dong Guo[1,2#], Yuntong Sun[1#], Junqi Wu[1#], Linchao Ding[1], Yiwen Jiang[1], Yadong Xue[1], Yongjun Ma[1], Fengtian Sun[1*]

[1]Department of Clinical Laboratory, Jinhua Central Hospital, Teaching Hospital of Mathematical Medicine College, Zhejiang Normal University, Jinhua, 321000, Zhejiang, China.

[2]Department of Orthopedic Surgery, Center for Orthopedic Surgery, The Third Affiliated Hospital of Southern Medical University, Guangzhou 510630, Guangdong, China.

[#]These authors contributed equally to this work.

*Corresponding author: Fengtian Sun: Email: jsdxsft@zjnu.edu.cn.

This Supplementary Materials includes:

Figure S1 to S14

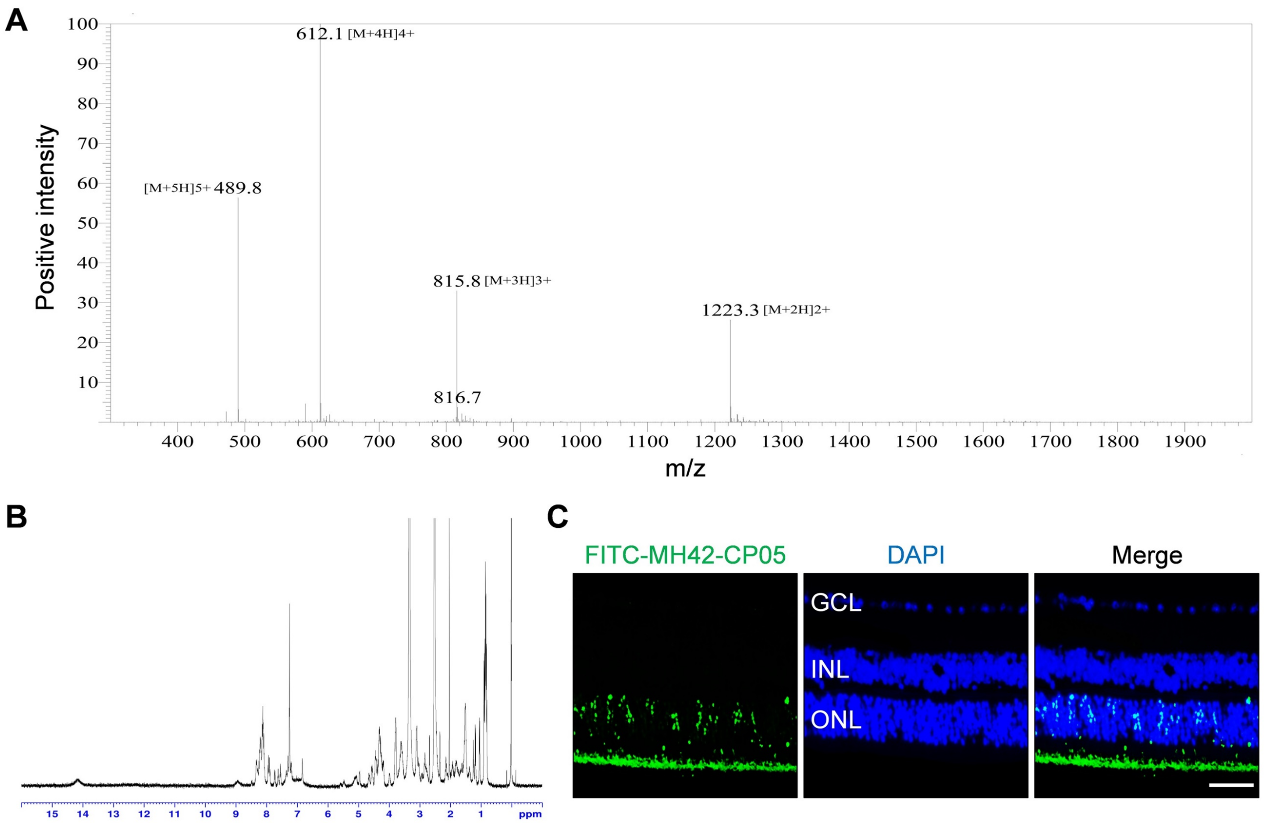

Figure S1. The characterization of MH42-CP05 fusion peptides. (A) Mass spectrum analysis of MH42-CP05 fusion peptides. (B) ^{1}H NMR spectrum analysis of MH42-CP05 fusion peptides. (C) The distribution of FITC-labeled MH42-CP05 fusion peptides in retinal tissues after intravitreal injection for 24 h. Scale bars, 100 μm. GCL, ganglion cell layer; INL, inner nuclear layer; ONL, outer nuclear layer.

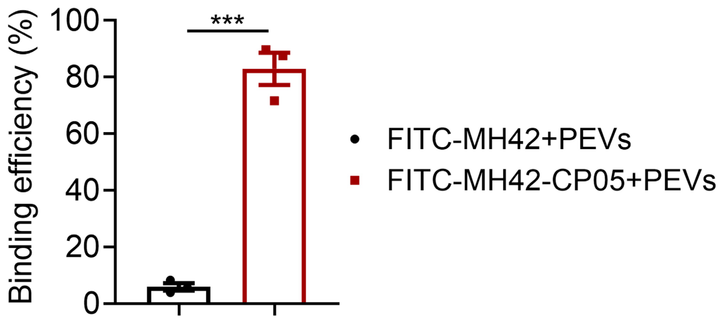

Figure S2. The binding efficiency of MH42-CP05 fusion peptides and MH42 peptides with PEVs (n=3). All data are presented as means ± SEM. ***$P < 0.001$.

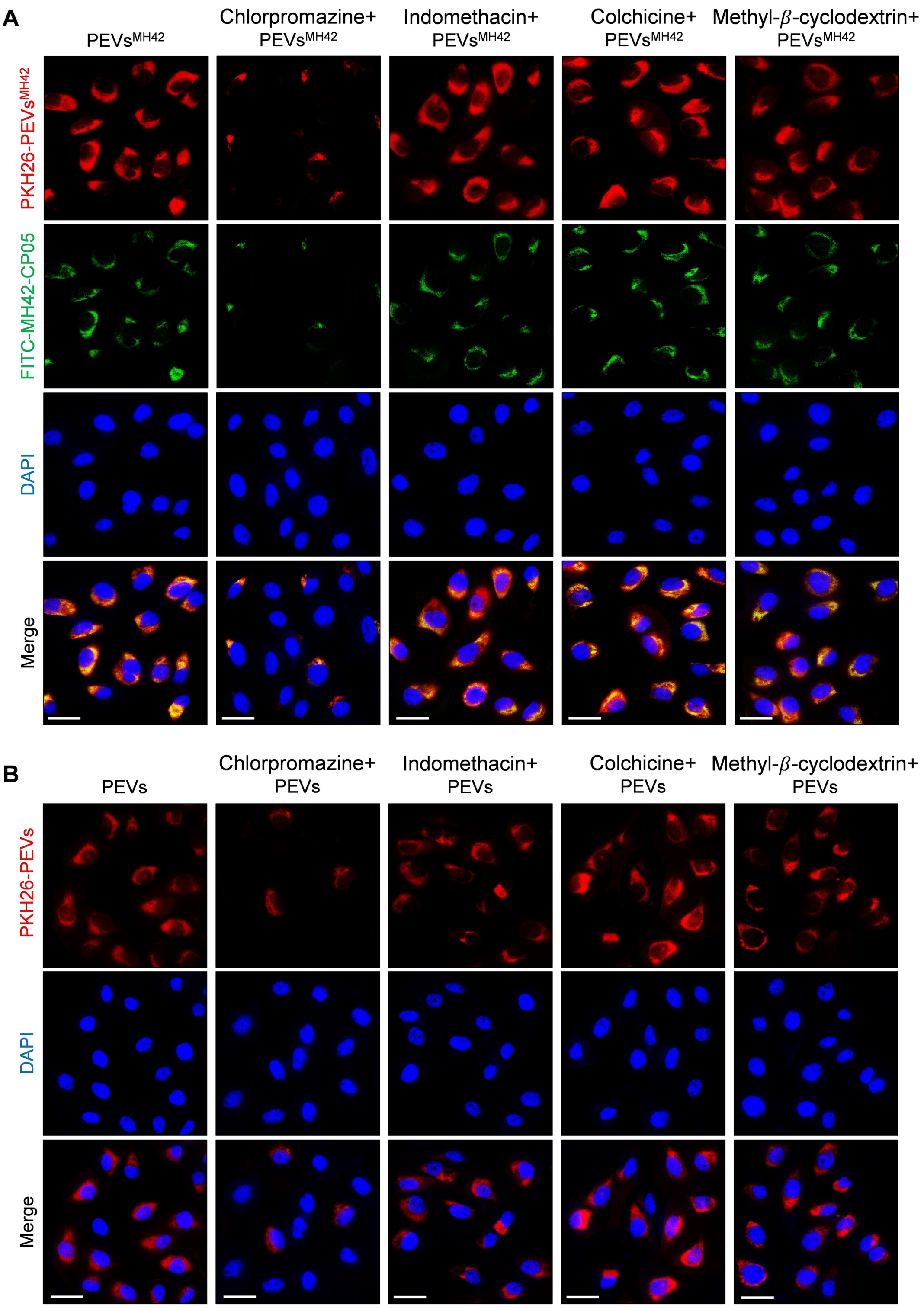

Figure S3. The internalization of PEVs^{MH42} (A) and PEVs (B) by 661W cells after pretreatment with endocytosis inhibitors. Scale bars, 25 μm.

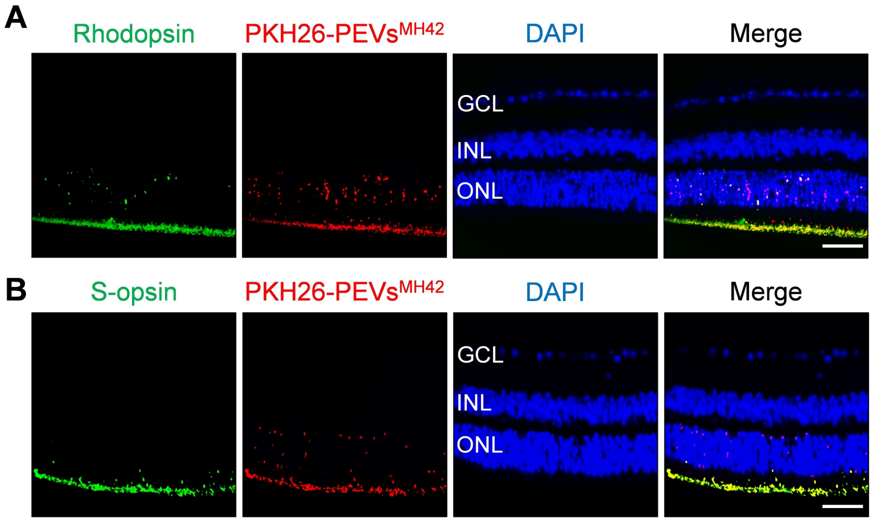

Figure S4. Immunofluorescence staining for the distribution of PKH26-labeled PEVs^{MH42} in rods (A) and cones (B). Scale bars, 100 μm. GCL, ganglion cell layer; INL, inner nuclear layer; ONL, outer nuclear layer.

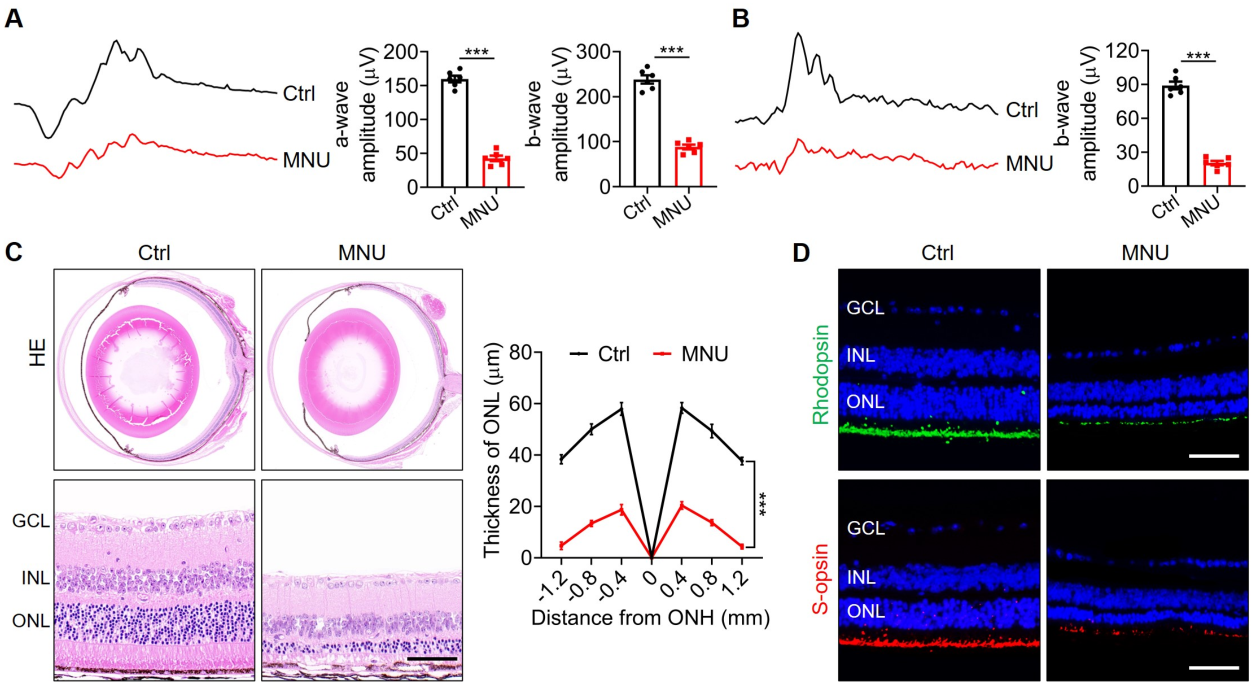

Figure S5. The identification of MNU-induced RD mouse model. (A) Representative scotopic ERG waveforms and the corresponding quantitative analysis of scotopic a-wave and b-wave amplitudes (n=6). (B) Representative photopic ERG waveforms and the corresponding quantitative analysis of photopic b-wave amplitude (n=6). (C) HE staining of retinal tissues and the corresponding quantitative analysis of ONL thickness (n=3). Scale bars, 100 μm. (D) Immunofluorescence staining for the retinal expression of rhodopsin and s-opsin. Scale bars, 100 μm. All data are presented as means ± SEM. ***$P < 0.001$. GCL, ganglion cell layer; INL, inner nuclear layer; ONL, outer nuclear layer.

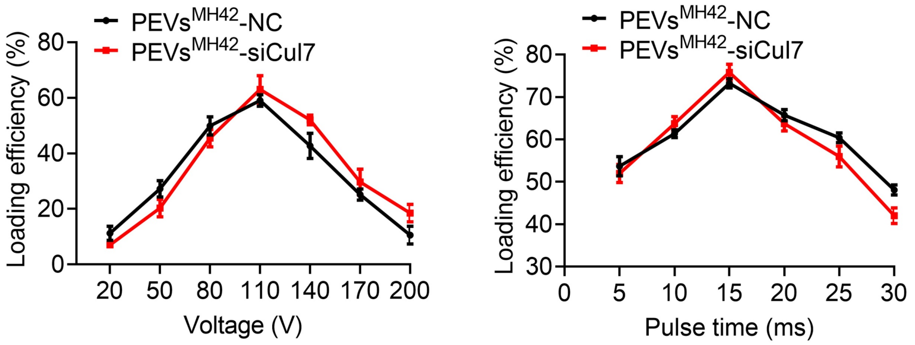

Figure S6. The loading efficiency of siCul7 and siRNA NC into PEVs^{MH42} through electroporation with different voltages and pulse times (n=3).

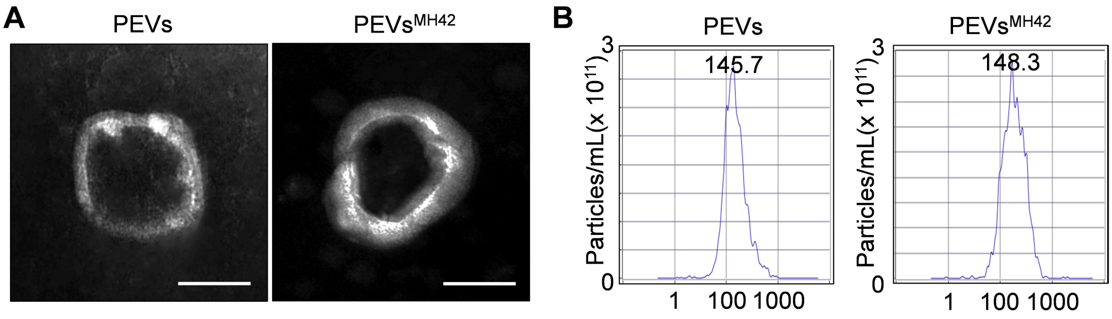

Figure S7. Characterization of PEVs and PEVs^{MH42}. (A) Representative TEM images of PEVs and PEVs^{MH42}. Scale bars, 100 nm. (B) NTA for the size distribution of PEVs and PEVs^{MH42}.

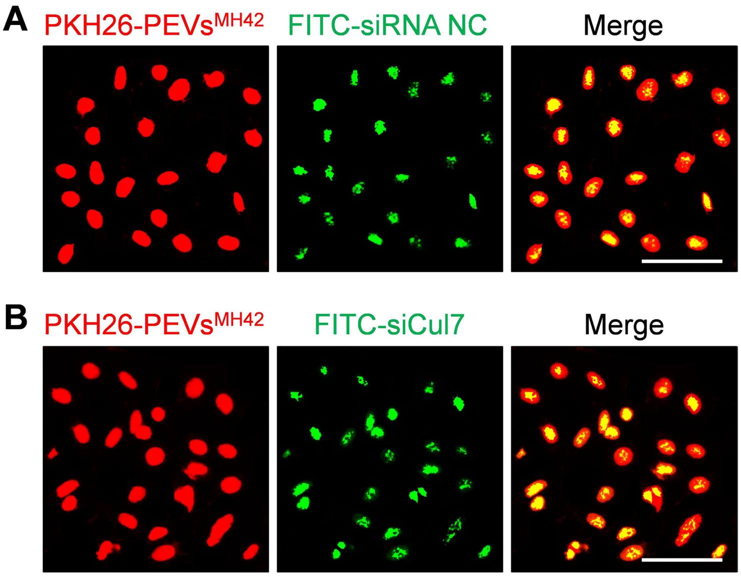

Figure S8. Representative confocal images of PEVs^{MH42}-NC (A) and PEVs^{MH42}-siCul7 (B). Yellow shows the co-localization of FITC-labeled siRNA NC or siCul7 with PKH26-labeled PEVs. Scale bar, 1 μm.

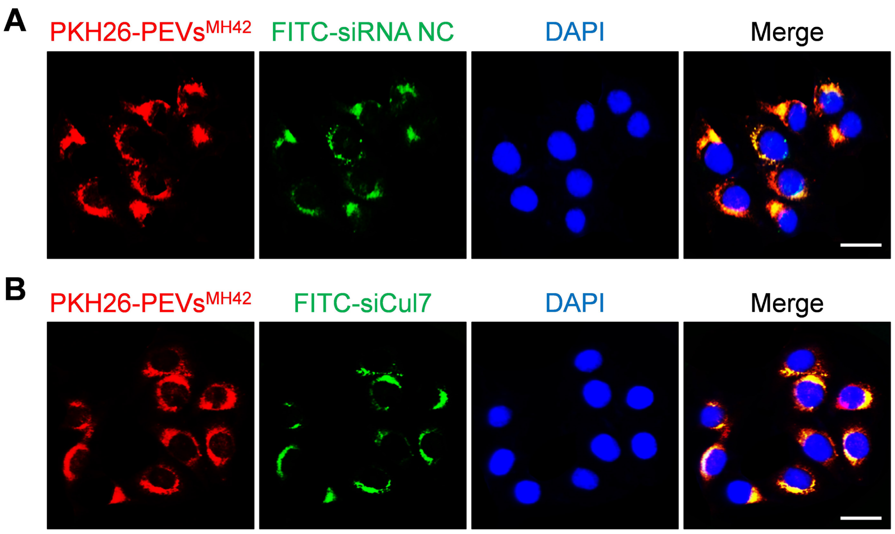

Figure S9. The internalization of PKH26-labeled PEVs^{MH42} and FITC-labeled siRNA NC (A) or siCul7 (B) by 661W cells after co-incubation for 48 h. Scale bars, 25 μm.

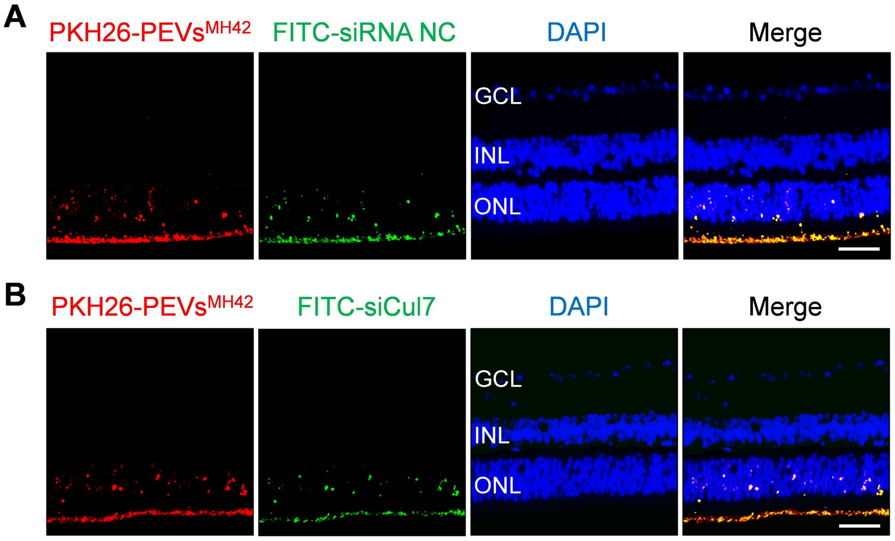

Figure S10. Tracing of PKH26-labeled PEVs^{MH42} and FITC-labeled siRNA NC (A) or siCul7 (B) in retinal tissues after intravitreal injection for 24 h. Scale bars, 100 μm. GCL, ganglion cell layer; INL, inner nuclear layer; ONL, outer nuclear layer.

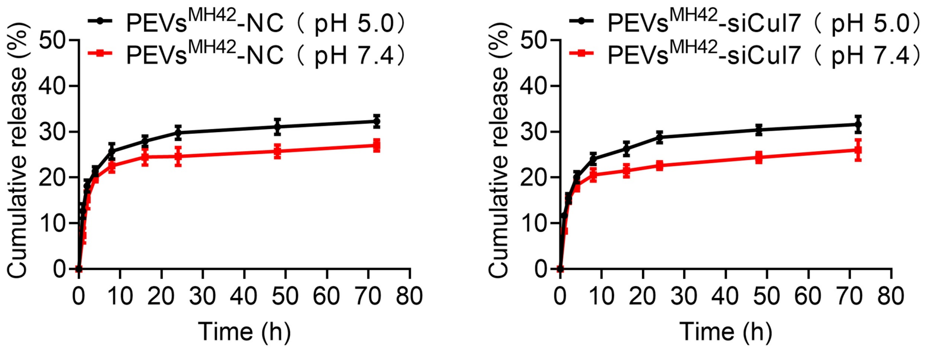

Figure S11. Release kinetic analysis of PEVs^{MH42}-NC and PEVs^{MH42}-siCul7 (n=3).

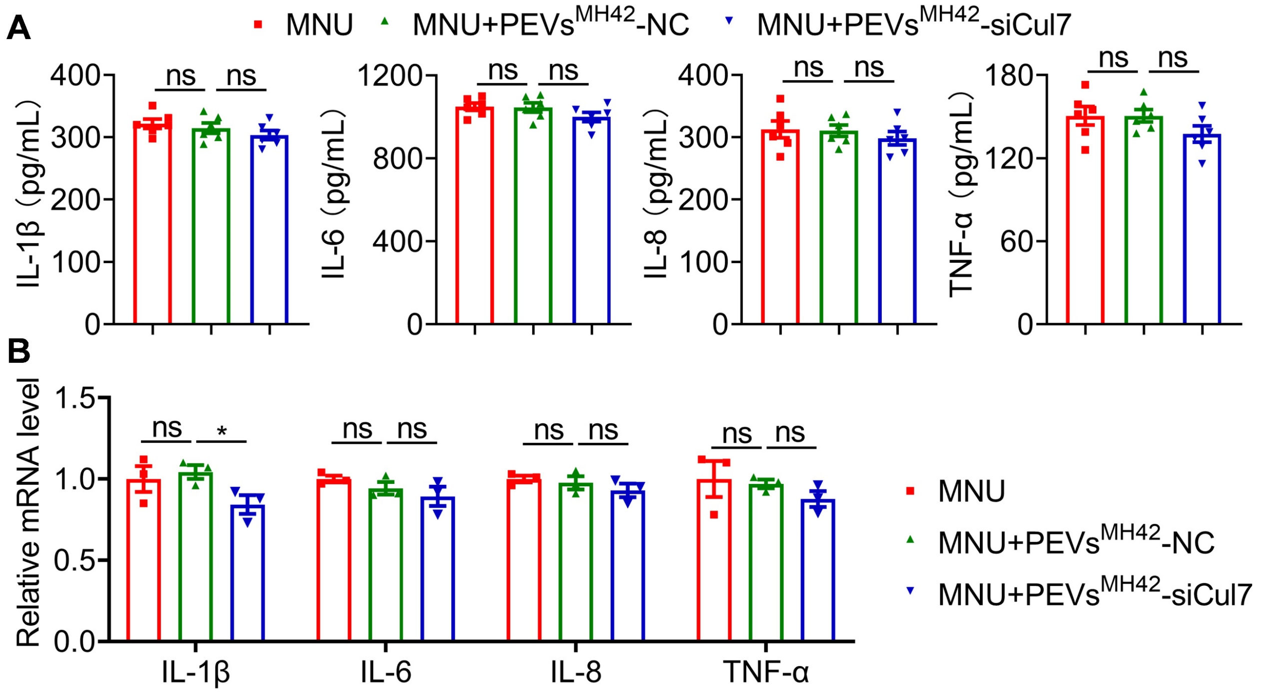

Figure S12. Evaluation of ocular inflammation in MNU-induced RD mice. (A) ELISA-based measurement of IL-1β, IL-6, IL-8, and TNF-α levels in the aqueous humor samples from MNU-induced RD mice. Each sample was pooled from aqueous humor of six eyes from six mice, and five biological repeats were quantified in each group. (B) qRT-PCR analysis for the mRNA levels of IL-1β, IL-6, IL-8, and TNF-α in retinal tissues (n=3). All data are presented as means ± SEM. ns, not significant, and $*P < 0.05$.

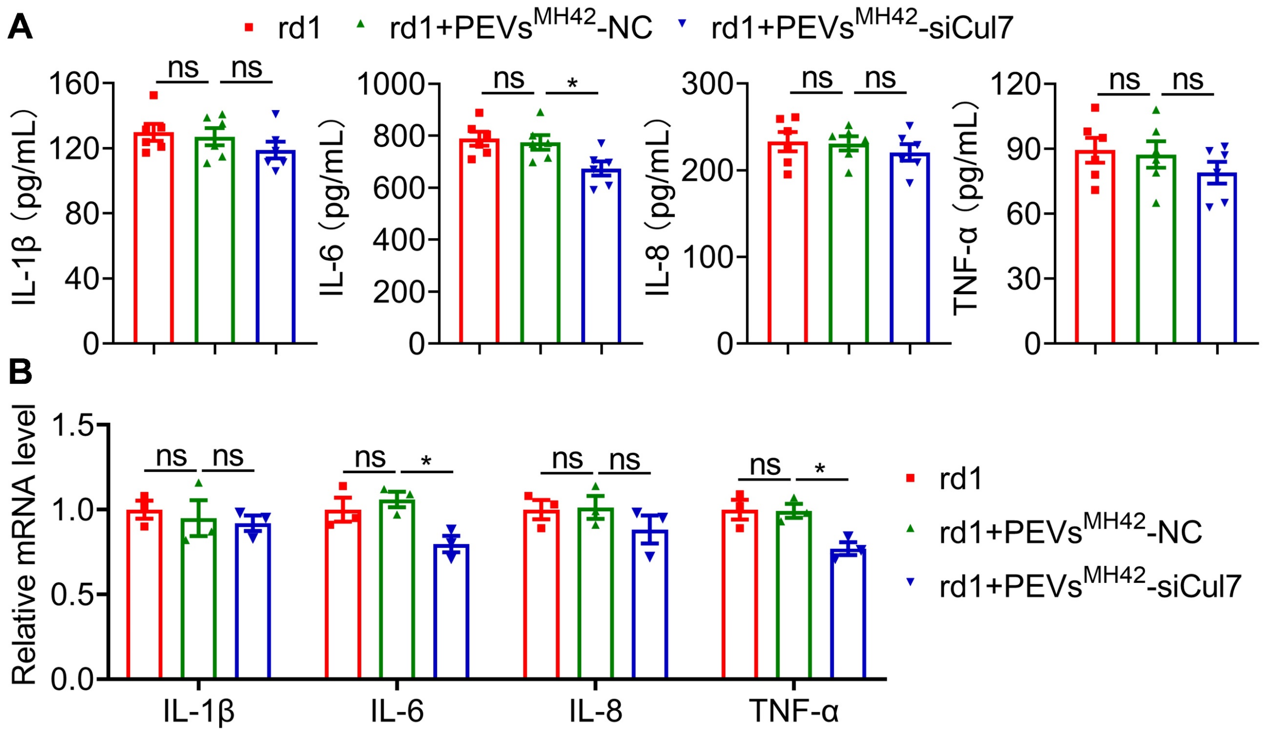

Figure S13. Evaluation of ocular inflammation in Pde6β$^{rd1/rd1}$ mutant mice. (A) ELISA-based measurement of IL-1β, IL-6, IL-8, and TNF-α levels in the aqueous humor samples from Pde6β$^{rd1/rd1}$ mutant mice. Each sample was pooled from aqueous humor of six eyes from six mice, and five biological repeats were quantified in each group. (B) qRT-PCR analysis for the mRNA levels of IL-1β, IL-6, IL-8, and TNF-α in retinal tissues (n=3). All data are presented as means ± SEM. ns, not significant, and *$P < 0.05$.

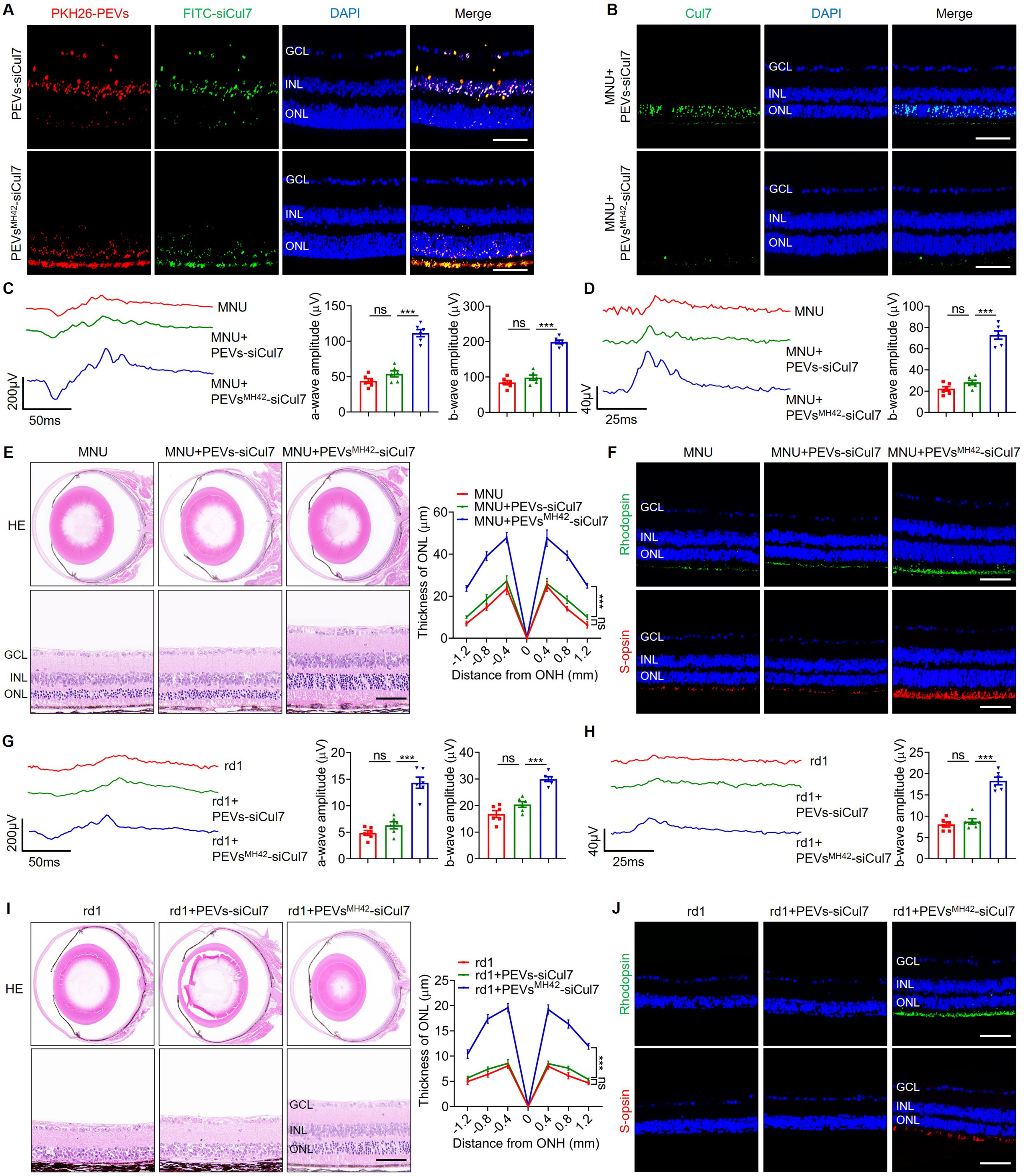

Figure S14. The targeting potential and therapeutic role of PEVs-siCul7 in vivo. (A) Tracing of PKH26-labeled PEVs-siCul7 or PEVs^{MH42}-siCul7 and FITC-labeled siCul7 in retinal tissues after intravitreal injection for 24 h. Scale bars, 100 μm. (B) Immunofluorescence staining for the retinal expression of Cul7 in MNU-induced RD mice treated with PEVs-siCul7 or PEVs^{MH42}-siCul7. Scale bars, 100 μm. (C) Representative scotopic ERG waveforms of MNU-induced RD mice and the corresponding quantitative

analysis of scotopic a-wave and b-wave amplitudes (n=6). (D) Representative photopic ERG waveforms of MNU-induced RD mice and the corresponding quantitative analysis of photopic b-wave amplitude (n=6). (E) Retinal HE staining of MNU-induced RD mice and the corresponding quantitative analysis of ONL thickness (n=3). Scale bars, 100 μm. (F) Immunofluorescence staining for the retinal expression of rhodopsin and s-opsin in MNU-induced RD mice. Scale bars, 100 μm. (G) Representative scotopic ERG waveforms of $Pde6\beta^{rd1/rd1}$ mutant mice and the corresponding quantitative analysis of scotopic a-wave and b-wave amplitudes (n=6). (H) Representative photopic ERG waveforms of $Pde6\beta^{rd1/rd1}$ mutant mice and the corresponding quantitative analysis of photopic b-wave amplitude (n=6). (I) Retinal HE staining of $Pde6\beta^{rd1/rd1}$ mutant mice and the corresponding quantitative analysis of ONL thickness (n=3). Scale bars, 100 μm. (J) Immunofluorescence staining for the retinal expression of rhodopsin and s-opsin in $Pde6\beta^{rd1/rd1}$ mutant mice. Scale bars, 100 μm. All data are presented as means ± SEM. not significant, and ***$P < 0.001$. GCL, ganglion cell layer; INL, inner nuclear layer; ONL, outer nuclear layer.
